# Mitochondrial Superoxide Production Decreases on Glucose-Stimulated Insulin Secretion in Pancreatic β Cells Due to Decreasing Mitochondrial Matrix NADH/NAD^+^ Ratio

**DOI:** 10.1089/ars.2019.7800

**Published:** 2020-09-10

**Authors:** Lydie Plecitá-Hlavatá, Hana Engstová, Blanka Holendová, Jan Tauber, Tomáš Špaček, Lucie Petrásková, Vladimír Křen, Jitka Špačková, Klára Gotvaldová, Jan Ježek, Andrea Dlasková, Katarína Smolková, Petr Ježek

**Affiliations:** ^1^ Department of Mitochondrial Physiology, No. 75, Institute of Physiology of the Czech Academy of Sciences, Prague, Czech Republic.; ^2^ Laboratory of Biotransformation, Institute of Microbiology of the Czech Academy of Sciences, Prague, Czech Republic.; ^3^ The Gurdon Institute, University of Cambridge, Cambridge, United Kingdom.

**Keywords:** mitochondrial superoxide generation, pancreatic β cells, glucose-stimulated insulin secretion, Complex I, NADH/NAD^+^ ratio, fluorescence lifetime imaging

## Abstract

***Aims:*** Glucose-stimulated insulin secretion (GSIS) in pancreatic β cells was expected to enhance mitochondrial superoxide formation. Hence, we elucidated relevant redox equilibria.

***Results:*** Unexpectedly, INS-1E cells at transitions from 3 (11 m*M*; pancreatic islets from 5 m*M*) to 25 m*M* glucose decreased matrix superoxide release rates (MitoSOX Red monitoring validated by MitoB) and H_2_O_2_ (mitoHyPer, subtracting mitoSypHer emission). Novel double-channel fluorescence lifetime imaging, approximating free mitochondrial matrix NADH_F,_ indicated its ∼20% decrease. Matrix NAD^+^_F_ increased on GSIS, indicated by the FAD-emission lifetime decrease, reflecting higher quenching of FAD by NAD^+^_F_. The participation of pyruvate/malate and pyruvate/citrate redox shuttles, elevating cytosolic NADPH_F_ (iNAP1 fluorescence monitoring) at the expense of matrix NADH_F_, was indicated, using citrate (2-oxoglutarate) carrier inhibitors and cytosolic malic enzyme silencing: All changes vanished on these manipulations. ^13^C-incorporation from ^13^C-L-glutamine into ^13^C-citrate reflected the pyruvate/isocitrate shuttle. Matrix NADPH_F_ (iNAP3 monitored) decreased. With decreasing glucose, the suppressor of Complex III site Q electron leak (S3QEL) suppressor caused a higher Complex I I_F_ site contribution, but a lower superoxide fraction ascribed to the Complex III site III_Qo_. Thus, the diminished matrix NADH_F_/NAD^+^_F_ decreased Complex I flavin site I_F_ superoxide formation on GSIS.

***Innovation:*** Mutually validated methods showed decreasing superoxide release into the mitochondrial matrix in pancreatic β cells on GSIS, due to the decreasing matrix NADH_F_/NAD^+^_F_ (NADPH_F_/NADP^+^_F_) at increasing cytosolic NADPH_F_ levels. The developed innovative methods enable real-time NADH/NAD^+^ and NADPH/NADP^+^ monitoring in any distinct cell compartment.

***Conclusion:*** The export of reducing equivalents from mitochondria adjusts lower mitochondrial superoxide production on GSIS, but it does not prevent oxidative stress in pancreatic β cells.

## Introduction

**FIG. 1.**
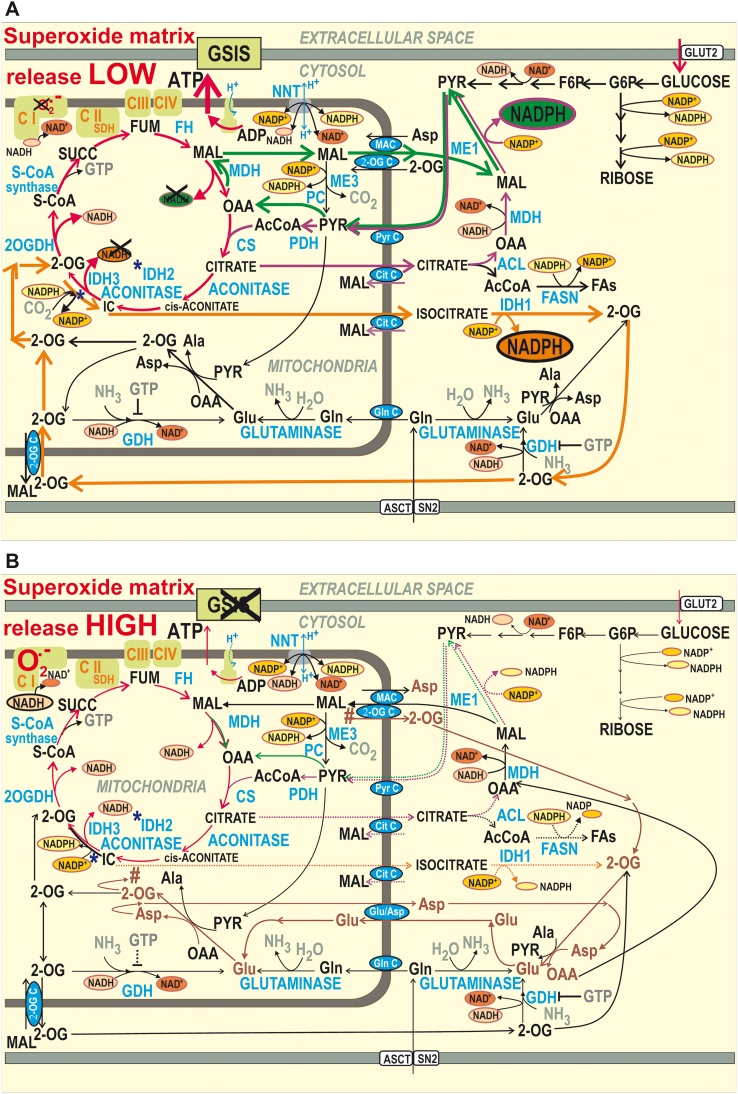
**Schematic illustrating the role of mitochondrial redox shuttles in pancreatic β cells on GSIS.** Mitochondrial redox shuttles are depicted by *arrows* of distinct colors: *dark green*—pyruvate/malate shuttle; *purple*—pyruvate/citrate shuttle; and *orange*—pyruvate/isocitrate shuttle. The malate-asparate shuttle is *brown*. **(A)** High glucose state, that is, stimulating insulin release. Mitochondrial pyruvate redox shuttles reportedly export reducing equivalents from the mitochondrial matrix into the cytosol on GSIS (34). Consequently, cytosolic NADPH should be hypothetically elevated at the expense of mitochondrial matrix NADH. The resulting elevated cytosolic NADPH has been reported to facilitate GSIS (52). Oxidative glucose metabolism leads to cytosolic ATP plus H_2_O_2_ elevation (48), thus initiating K_ATP_ channel-dependent GSIS (2, 52, 55). Glutamate dehydrogenase is depicted as producing glutamate (44); NNT is depicted to allow both reverse and forward mode (58). **(B)** Low glucose state: Instead of the redox shuttles cited earlier, the malate-aspartate shuttle (*brown*) may play a dominant role in mediating metabolic fluxes at low, nonstimulating, glucose concentration, which results in the transfer of redox equivalents of NADH into the mitochondrial matrix. Note that both the malate-aspartate carrier and 2OGC conduct the opposite fluxes within the malate-aspartate shuttle compared with the three pyruvate redox shuttles acting on GSIS. ^#^Continuation of flux within the malate-aspartate shuttle. *Indicates IDH2 reaction. 2OG, 2-oxoglutarate; 2-OG C, 2-oxoglutarate carrier; 2OGDH, 2-oxoglutarate dehydrogenase; AcCoA, acetyl-CoA; ACL, ATP-citrate lyase; ASCT, alanine serine cysteine transporter; CS, citrate synthase; CitC, citrate carrier; FA, fatty acid; FASN, fatty acid synthase; FH, fumarate hydratase; GDH, glutamate dehydrogenase; Gln C, glutamine carrier; GLUT, glucose transporter; GSIS, glucose-stimulated insulin secretion; K_ATP_, ATP-sensitive potassium; IDH2, isocitrate dehydrogenase 2; MAC, malate aspartate carrier; MDH, malate dehydrogenase; ME, malic enzyme; NNT, nicotinamide nucleotide transhydrogenase; PC, pyruvate carboxylase; PDH, pyruvate dehydrogenase; PyrC, pyruvate carrier; S-CoA, succinyl coenzyme A; SDH, succinate dehydrogenase; SN2, system N transporter 2; SUCC, succinate. Color images are available online.

The bioenergetics of pancreatic β cells was considered to be relatively well understood (10, 32, 33). Mitochondria of β cells represent the perfect glucose sensor, participating in glucose-stimulated insulin secretion (GSIS) (2, 20, 28, 32, 33, 52, 55). Glucose sensing was considered to be exclusively coupled with insulin exocytosis by elevated ATP, stemming from augmented glucose metabolism and oxidative phosphorylation (OXPHOS). However, recently we revealed that, in addition to ATP, also a parallel redox signaling from NADPH oxidase 4 (NOX4) is essential for GSIS (48). In β cells, the insulin-independent glucose transporter GLUT2 (in rodents) allows cell glucose equilibration with plasma glucose levels. Glycolysis is 100% restricted to the pyruvate dehydrogenase (PDH) and pyruvate carboxylase reaction followed by OXPHOS (Fig. 1A, B) (2, 10, 32, 33, 52, 55). The concomitantly increased ATP/ADP ratio within the sub-plasma-membrane cytosolic microdomain was believed to be sufficient to induce the closure of ATP-sensitive potassium (K_ATP_) channels, thus depolarizing the plasma membrane and activating voltage-gated L-type Ca^2+^channels (Ca_L_) (2, 52, 55). However, we demonstrated that a parallel H_2_O_2_ burst is essentially required to close the K_ATP_ channel, together with ATP. Any single component (sole ATP or sole H_2_O_2_) is not sufficient to stimulate insulin secretion (48). The resulting Ca^2+^ influx elevates cytosolic Ca^2+^ concentration and stimulates Ca^2+^-dependent exocytosis of insulin-containing granules.

**Innovation**

The concomitant decline in superoxide/H_2_O_2_ release to the mitochondrial matrix was documented by three mutually validated methods to be dependent on the operation of three redox shuttles on glucose-stimulated insulin secretion in INS-1E cells. Fluorescent iNAP3 and iNAP1 probes indicated a decrease in matrix and an increase in cytosolic NADPH_F_. The latter was confirmed by novel double-channel fluorescence lifetime imaging, which together with elevated β-hydroxybutyrate indicated increasing matrix NAD^+^_F_ at lowered NADH_F_, hence decreasing NADH_F_/NAD^+^_F_ ratios. Inspections of superoxide formation sites by S3(1)QEL identified the Complex I I_F_ site as the one that decreased superoxide formation when there was a diminished NADH_F_/NAD^+^_F_ ratio.

β Cell mitochondria represent a highly relevant source of reactive oxygen species (ROS) (1, 16, 30, 31, 39). Superoxide is primarily formed and converted to H_2_O_2_ by manganese superoxide dismutase (MnSOD) or copper-zinc superoxide dismutase (CuZnSOD) in the mitochondrial matrix or intermembrane space, respectively (8, 11, 30, 31). The resulting H_2_O_2_ may also serve as a signaling molecule (50). Indeed, insulin release is also stimulated due to the elevated superoxide formation by β-like oxidation of branched-chain keto-acids in mitochondria, wherein superoxide is converted to “signaling” H_2_O_2_, diffusing up to the K_ATP_ channel to aid its closure (48). However, whether such matrix redox burst exists in glucose metabolism is not yet resolved.

Concomitantly, three described redox shuttles export reducing equivalents from the mitochondrial matrix into the cytosol on GSIS in pancreatic β cells (34): (i) the pyruvate/malate, (ii) pyruvate/citrate, and (iii) pyruvate/isocitrate shuttle (Fig. 1A, B) (35). Their operation should elevate cytosolic NADPH production at the expense of matrix NADH. The first two shuttles deliver substrates for the cytosolic malic enzyme (ME1) (34), and the third one delivers substrates for isocitrate dehydrogenase 1 (IDH1) (22). ME1 and IDH1 subsequently produce NADPH. The pyruvate carrier (PyrC) and 2-oxoglutarate carrier (2OGC) and matrix malate dehydrogenase (MDH) are essential for the pyruvate/malate shuttle (34), PyrC and citrate carrier (CitC) and ATP-citrate lyase represent the pyruvate/citrate shuttle (34) and 2OGC and CitC plus matrix NADPH-consuming isocitrate dehydrogenase 2 (IDH2), acting in the reductive carboxylation mode (59), are essential for the pyruvate/isocitrate shuttle (Fig. 1A) (22, 34). Invariably, these redox shuttles presumably elevate cytosolic NADPH when active. GSIS is then slightly facilitated by the resulting NADPH increase (22, 35, 52) that adds to the production of pentose-phosphate pathway (PPP) supplying NOX4 (48). For each molecule of malate or citrate/isocitrate exported from the mitochondrial matrix by the respective shuttle, one molecule of NADH should be missing for Complex I, since MDH or isocitrate dehydrogenase 3 (IDH3) (otherwise producing NADH) cannot use their substrates. Thus, mitochondrial matrix NADH formation is predicted to be lower and the matrix NAD^+^ is assumed to accumulate on GSIS. In this work, we aimed at experimentally supporting this scheme (Fig. 1A; Hypothesis I).

Mitochondrial superoxide formation takes place as an inevitable side reaction of electrons with oxygen within the sites of mitochondrial respiratory chain Complexes I and III, by dysfunctional Complex II, and sites of matrix 2-oxoacid dehydrogenases and cytosolic-oriented glycerol phosphate dehydrogenase (8). In total, 11 different redox sites have been recognized to generate superoxide in mitochondria (8). Under specific conditions, superoxide formation at the Complex I site I_ubiquinone_ (I_Q_) or Complex III outer (III_Qo_) site increases with increasing protonmotive force Δ*p* (18, 25, 29, 45, 57). Consequently, the attenuation of mitochondrial superoxide formation by active OXPHOS can occur on the re-entry of protons into the matrix *via* the F_O_ membrane sector of the ATP-synthase (18). Superoxide production also diminishes/rises on the acceleration/retardation of cytochrome *c* shuttling, respectively (the latter independently of Δ*p*) (21, 29, 45, 50), and it depends on ubiquinone homeostasis, or the rate of the reverse electron transport (8, 13, 65).

Finally, superoxide can be generated at the Complex I flavin (I_F_) site, which resides close to the flavin-binding site at which NADH is oxidized (40, 65). Here, the increased substrate pressure *S*, defined as the NADH/NAD^+^ ratio, induces substantial superoxide production (40, 65). Since there is an increased substrate load in pancreatic β cells on GSIS, faster superoxide production could be predicted. However, the activity of the shuttles mentioned earlier may modulate the Complex I substrate pressure in a manner that is still unpredictable.

Questions of whether superoxide formation increases or decreases on GSIS have already been raised. Opinion was ambiguous. With extensive glucose depletion, the effect of substrate load may overcome the suppressing role of H^+^ returning *via* the ATP-synthase. Accordingly, increasing mitochondrial ROS on GSIS has been observed (5, 41, 56). Increasing ATP with decreasing ADP was predicted to diminish mitochondrial ROS formation (23). Indeed, it has been demonstrated for rat pancreatic islets (PIs) that stimulation with glucose reduced the mitochondrial oxidation of roGFP2-Orp1 (15). The superoxide-suppressing role of H^+^ re-entry *via* the F_O_ sector of the ATP-synthase at a higher intensity of OXPHOS might predominate (37). Also, total reducing equivalents were reported to be increased on GSIS (47). However, the addition of pyruvate alone to PIs increased cellular NADPH without increasing NADH, in contrast to glucose that increased both (53).

To elucidate changes associated with redox homeostasis on GSIS, we have introduced several novel methods to assess nicotinamide nucleotides in the separate compartments of the mitochondrial matrix and cytosol. Moreover, confocal microscopy monitoring with selective fluorescence probes enabled us to study mitochondrial matrix superoxide and H_2_O_2_ generation on the sudden addition of glucose. This approach was validated by using a MitoB probe to monitor ROS accumulation (14, 36, 67, 68). Therefore, by independent means, we provide several lines of unequivocal evidence for a diminished mitochondrial superoxide production on GSIS due to the elevated operation of redox shuttles.

## Results

### GSIS increases mitochondrial respiration and membrane potential in INS-E cells

**FIG. 2.**
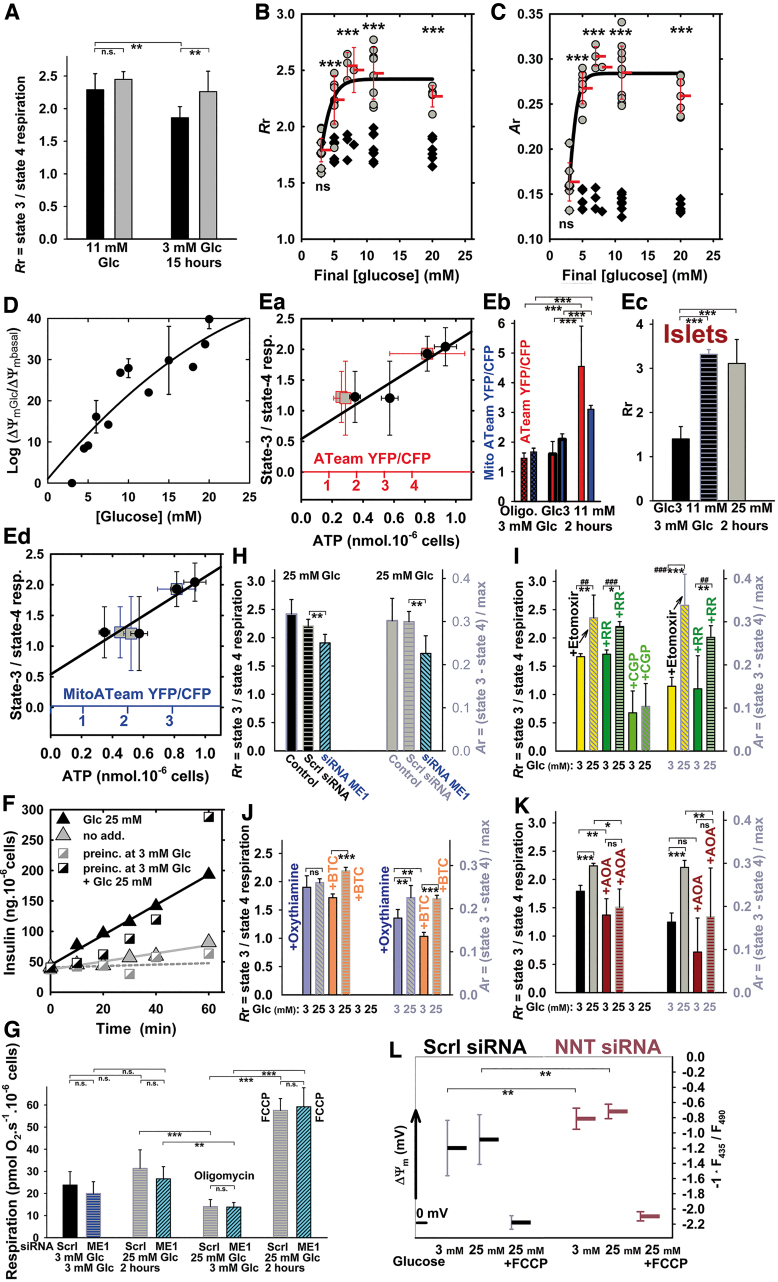
**Respiration, bioenergetic profile,** Δ*Ψ*_**m**_**, ATP levels, and insulin secretion in INS-1E cells. (A)** Phosphorylating to nonphophosporylating respiration ratio (*R*r) before (*black bars*) and after (*gray bars*) glucose addition to reach 25 m*M* final concentration. INS-1E cells were cultured in 11 m*M* glucose (“11 m*M*”) or preincubated in cell culture medium with 3 m*M* glucose (“3 m*M*”) for 15 h. ANOVA (*n* = 13–15): **p < 0.05. **(B, C)** Glucose-dose dependence of phosphorylating/nonphosphorylating respiratory rate ratio *R*r **(B)** and the parameter *A*r **(C)**. INS-1E cells were preincubated in KRH^BSA^ containing 3 m*M* glucose for 1 h. *Black symbols* represent parameters calculated relatively to the respective respiration at 3 m*M* glucose, whereas *gray symbols* at 20 m*M* glucose. Endogenous, that is, phosphorylating respiration rates were always recorded before glucose additions to desired concentrations, and after these additions. This was followed by the addition of 1 μ*M* oligomycin, but oligomycin was also added to KRH^BSA^ with 3 m*M* glucose in parallel runs. In this way, parameter *R*r was calculated for situations before (*black symbols*) and after glucose addition (*gray symbols*). FCCP was adjusted (titrated) for maximum response to estimate the maximum (uncoupled) respiration for each glucose concentration, and data were used to calculate the parameter *A*r (phosphorylating minus nonphosphorylating respiration rate divided by the maximum respiration rate with FCCP). Student's *t*-test: ***p < 0.001. **(D, L)** Mitochondrial membrane potential (Δ*Ψ*_m_) estimated by using TMRE **(D)** and JC-1 **(L)**. **(D)** Δ*Ψ*_m_ is plotted against final glucose concentrations as ratios of TMRE fluorescence after (Δ*Ψ*_m_Glc) and before (Δ*Ψ*_m_basal) glucose addition on a logarithmic scale; **(L)** JC-1-estimated Δ*Ψ*_m_ is displayed in a diagram for 3 and 25 m*M* glucose in cells transfected with a scrambled (“Scrl”) siRNA or NNT siRNA. ANOVA (*n* = 8): **p < 0.02. **(E)** Correlations between ATP levels assayed using bioluminescence (*black points*) or cytosolic **(Ea**; *red points*; *red* x-axis**)** and mitochondrial matrix ATeam FRET-sensor **(Ed**; *blue points*; *blue* x-axis**)** and phosphorylating to nonphophosporylating respiration ratios *R*r—INS-1E cells were preincubated at varying glucose in cell culture medium without pyruvate for 2 h and, subsequently, respiration and ATP levels were measured. Confidence for the correlations was 95%. **(Eb)** Ratios of YFP and CFP emission of the ATeam FRET sensors are plotted before and after transition from 3 to 20 m*M* glucose (same color coding as above). ANOVA (*n* = 33–47): ***p < 0.001. **(Ec)** Parameter *R*r for respiration of PIs—assayed by using the Seahorse apparatus, in which irreproducible results are obtained with FCCP, hence *A*r cannot be derived. **(F)** Time courses for insulin secretion. Secreted insulin levels are shown after glucose addition (25 m*M* final concentration) to INS-1E cells cultured in 11 m*M* glucose (*black triangles*) or preincubated in standard cell culture medium containing 3 m*M* glucose for 2 h (*black squares*). *Gray symbols*: time courses without glucose addition. In addition, cells were preincubated in KRH buffer for 5 min before the ELISA insulin assay in KRH. **(G)** Effect of ME1 silencing on endogenous respiration rates (*n* = 6) with 3 and 25 m*M* glucose and nonphosphorylating respiration (“oligomycin”; 1 μ*M* oligomycin) and maximum respiration (“FCCP,” adjusted for maximum response) with 25 m*M* glucose; or **(G–K)** effects of ME1 silencing and metabolic or transport inhibitors **(I–K)** on respiratory parameters calculated as described earlier—*R*r (left y-axis) and *A*r (right y-axis). For the original respiration data and their replicates, see Supplementary Figures S1 and S2. Ruthenium red (7 μ*M*), etomoxir (100 μ*M*), 10 μ*M* CGP37157, oxythiamine (40 μ*M*), BTC (10 m*M*), and AOA (4 m*M*) were present as indicated. ANOVA (*n* = 4–6): *p < 0.1; **p < 0.05; ***p < 0.001; or Student's *t*-test for chosen pairs: ^##^*p* < 0.05; ^###^*p* < 0.001. ANOVA, analysis of variance; AOA, aminooxyacetic acid; BSA, bovine serum albumin; BTC, 1,2,3-benzene-tricarboxylate; FCCP, 4-(trifluoromethoxy)phenylhydrazone; KRH, Krebs-Ringer HEPES buffer; ME1, cytosolic malic enzyme; ns, nonsignificant; PI, pancreatic islet; siRNA, small interfering RNA; TMRE, tetramethylrhodamine ethyl ester. Color images are available online.

Unlike with initial 11 m*M* glucose, INS-1E cells preincubated with 3 m*M* glucose for 2 or 15 h in cultivation medium responded to the addition of glucose (25 m*M* final concentration) by significantly increasing their respiration in the cultivation medium, phosphorylating**/**nonphosphorylating respiration ratio (*R*r, Fig. 2A, B), parameter *A*r (Fig. 2C), and mitochondrial inner membrane potential Δ*Ψ*_m_ (Fig. 2D). A strict correlation was found between the ratio *R*r and total ATP levels (Fig. 2Ea) or local ATP in the cytosol (excluding ATP signal from insulin granules; Fig. 2Ea, Eb) and mitochondrial matrix ATP (Fig. 2Eb, Ed), assayed by using the respective FRET-based ATeam biosensors. Notably, *R*r represents valid estimates of ATP synthesis (OXPHOS activity) required for GSIS (Fig. 2F). In INS-1E cells (Fig. 2B,C) and isolated PIs (Fig. 2Ec), an array of phosphorylating states exists within a sharp hyperbolic increase *versus* glucose concentration in *R*r (Fig. 2B) (60) or *A*r (a fraction of respiration used for ATP synthesis calculated as phosphorylating minus nonphosphorylating respiration normalized to maximum, that is, uncoupled respiration; Fig. 2C). Both were assayed in Krebs-Ringer HEPES buffer containing bovine serum albumin (KRH^BSA^) after a 1-h preincubation in KRH^BSA^ containing 3 m*M* glucose. In cells, a steep increase was found between 3 and 8 m*M* with the half-maxima of *R*r and *A*r at ∼3.5 and 4 m*M* glucose, respectively, and saturation above 8 m*M* glucose (Fig. 2B, C).

Silencing the cytosolic malic enzyme, ME1 insignificantly inhibited respiration at low and high glucose, whereas H^+^-leak-driven (nonphosphorylating) or maximum (uncoupled) respiration rates were equal (Fig. 2G and Supplementary Fig. S1Aa, Ab). Comparing oxygraph record pairs separately, *R*r decreased by ∼15% and *A*r by ∼25% (Fig. 2H). Etomoxir, an irreversible inhibitor of carnitine palmitoyltransferase-1 that blocks fatty-acid β-oxidation (28), did not affect the *R*r (*A*r) elevations on transitioning to high glucose (Fig. 2I and Supplementary Fig. S2Aa, Ab). Similarly, inhibiting Ca^2+^ uptake into mitochondria with ruthenium red had no effect on *R*r and *A*r (Fig. 2I), neither on respiration (Fig. S2Ba, Bc). However, elimination of Ca^2+^efflux through the Ca^2+^/Na^+^-antiporter using CGP37157 inhibited phosphorylating respiration (down to 30–40%), H^+^-leak-driven respiration, and elevations in ATP synthesis (Fig. 2I and Supplementary Fig. S2Bb, Bc). Silencing of nicotinamide nucleotide transhydrogenase (NNT) in combination with Δ*Ψ*_m_ monitoring suggested the existence of forward NNT mode (NADH-, NADP^+^- plus Δp-consuming, producing NAD^+^ and NADPH) at both 3 and 25 m*M* glucose, since Δ*Ψ*_m_ increased on NNT silencing (Fig. 2L); cf. Santos *et al.* (58).

Blockage of PPP with oxythiamine even elevated *R*r and *A*r with 3 m*M* glucose (Fig. 2J). Inhibition of CitC with 1,2,3-benzene-tricarboxylate (BTC) did not significantly affect respiration (Supplementary Fig. S1Ca, Cb), *R*r, or *A*r (Fig. 2J). Amino-transferase/transaminase inhibitor aminooxyacetic acid (AOA) inhibited respiration by 60%, maximum respiration under 10%, and decreased *R*r and *A*r (Fig. 2K and Supplementary Fig. S2Ca, Ce). Maximum respiration was inhibited under 14% (plus the glucose-induced respiration rise) when glycolysis was blocked with bromopyruvate.

### Mitochondrial matrix superoxide release is attenuated on transition to high glucose

**FIG. 3.**
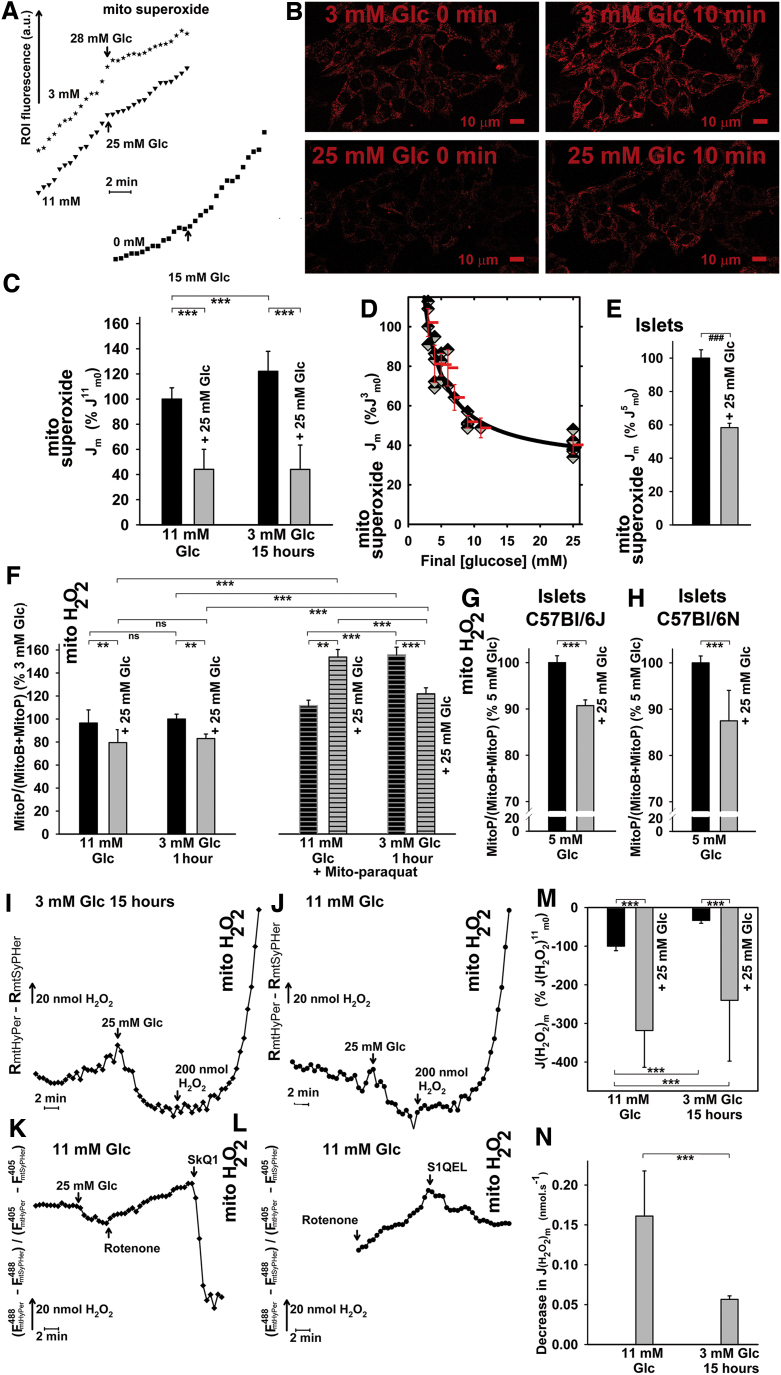
**Release of superoxide and H_2_O_2_ into the mitochondrial matrix of INS-1E cells on GSIS.** INS-1E cells **(A–D**) were assayed for surplus mitochondrial matrix superoxide release (over MnSOD consumption) by using confocal microscopy monitoring **(B)** of MitoSOX Red fluorescence (see also Supplementary Fig. S3G). PIs were assayed similarly **(E)** by using MitoSOX Red spectra scanning. *Black columns*: values before GSIS; *gray columns*: values after GSIS. **(A)** Representative traces—*J*_m_ rates were determined from the slopes of linearized traces, such as illustrated, of increasing MitoSOX Red integral fluorescence encompassing mitochondrial ROI, plotted *versus* time for each series of the corresponding confocal images (18, 28, 29). When indicated, INS-1E cells were cultured in 11 m*M* glucose, or preincubated in medium containing 3 m*M* glucose for 15 h. Alternatively, cells were preincubated in KRH medium containing bicarbonate “0 m*M*.” Final glucose levels after a glucose addition are indicated by *arrows*. **(B)** Representative confocal images for MitoSOX Red assay carried out separately for 3 and 25 m*M* glucose. **(C)** Quantification of mitochondrial superoxide production rates *J*_m_ for cells cultured in medium containing 11 m*M* glucose (“11 m*M* Glc”) or preincubated in cell culture medium containing 3 m*M* glucose (“3 m*M* Glc 15 h”). *J*_m_ rates were normalized to average *J*_m_ rates obtained in cells cultured in 11 m*M* glucose before GSIS (*J*^11^_m0_). ANOVA (*n* = 11; *n* = 6 for 3 m*M* 15 h): ***p < 0.001. **(D)** Decrease in mitochondrial matrix-released superoxide—dose response related to the final glucose concentration ([glucose]) performed by using MitoSOX Red confocal microscopy monitoring. The higher the final glucose concentration reached, the higher the decrease in *J*_m_ rates on glucose addition. **(E)** Isolated mouse PIs: *J*_m_ rates before (*black column*) and 15 min after glucose addition (*gray column*) were derived from changes in MitoSOX Red spectra after glucose addition to isolated mouse PIs (cf. Supplementary Fig. S3H). Student's *t*-test (*n* = 3): ^###^*p* < 0.001. **(F–H)** MitoP/(MitoB+MitoP) ratios reflecting matrix ROS accumulated after 2 h in INS-1E cells or PIs isolated from the indicated mouse strains. Ratios were normalized to values obtained for incubations in medium with 3 m*M* glucose. Mito-paraquat, 20 μ*M*. ANOVA (*n* = 4): **p < 0.05; ***p < 0.001. **(I–N)** Mitochondrial matrix H_2_O_2_ release. *J*_m_^H2O2^ rates were assessed by mito-HyPer fluorescence confocal microscopy monitoring, whereas pH changes were accounted for by mito-SypHer. The differences in these records were taken as being proportional to the net mitochondrial matrix H_2_O_2_ release. **(I–K)** Representative differential time courses are illustrated for cells preincubated in 3 m*M* glucose **(I)** or cultured in medium with 11 m*M* glucose **(J)**, and after the addition of 20 μ*M* rotenone **(K)** or 1 μ*M* S1QEL **(L)**. Relative **(M)** and approximate absolute **(N)** quantifications of the resulting *J*_m_^H2O2^ rates are shown. ANOVA (*n* = 3–6): ***p < 0.001. MnSOD, manganese superoxide dismutase; ROI, regions of interests; ROS, reactive oxygen species; S1QEL, suppressor of complex 1 site Q electron leak. Color images are available online.

First, we positively validated the method employing the time-lapsed acquisition of MitoSOX Red fluorescence confocal microscopy (Supplementary Fig. S3). Therefore, we can interpret the MitoSOX Red fluorescence elevation to be a genuine measure of the increased superoxide release into the mitochondrial matrix. In INS-1E cells routinely cultured with 11 m*M* glucose, there is a nonzero mitochondrial matrix superoxide release (Fig. 3A–D and Supplementary Fig. S3B). Unexpectedly, a rather sharp decrease in superoxide release into the matrix was observed after the addition of glucose (25 m*M* final) (Fig. 3A and Supplementary Fig. S3G). The corresponding estimates of superoxide release into the matrix *J*_m_ decreased and remained low (Fig. 3A, C). The *J*_m,_ rates represent slopes of increase in integral fluorescence (Fig. 3B and Supplementary Fig. S3G). These values accounted for ∼45% of *J*_m_ values before glucose addition (Fig. 3C). Such a drop could not originate from the decreasing Δ*Ψ*_m_, even if MitoSOX Red fluorescence depended on Δ*Ψ*_m_ (which is not the case, see Supplementary Fig. S3). This is because Δ*Ψ*_m_ increases after glucose addition (Fig. 2D) (60). Moreover, the data were qualitatively similar on NNT silencing (Supplementary Fig. S2Dc).

MnSOD (Supplementary Fig. S4A) and total superoxide dismutase (SOD) activities (Supplementary Fig. S4B), measured in parallel, were constant. We also preincubated INS-1E cells in medium containing only 3 m*M* glucose for 15 h (Fig. 3A, C) or 2 h (Fig. 3D and Supplementary Fig. S3E–G), which also exhibited diminished *J*_m_ after glucose addition (Fig. 3A, C and Supplementary Fig. S3E–G), with a sharp decline between 4 and 9 m*M* glucose (Fig. 3D), thus precisely matching glucose-dose dependencies for *R*r and *A*r (Fig. 2B, C). This was not observed in completely glucose-depleted cells (Fig. 3A).

To demonstrate the independence of MitoSOX Red responses on the plasma membrane potential (Δ*Ψ*_p_), we determined the influence of Δ*Ψ*_p_ changes on *J*_m_ rates. Despite having Δ*Ψ*_p_ depolarization induced with glibenclamide before the addition of glucose or blocked with cromakalim, the obtained responses to glucose were equal (Supplementary Fig. S3E, F). The basal *J*_m_ rates were on average 1.7 ± 0.1 times and 1.2 ± 0.3 times higher than for INS-1E cells preincubated for 25 h (Fig. 3A, C) and 2 h (Supplementary Fig. S3E, F) with 3 m*M* glucose, respectively. The additions of 25 m*M* glucose caused these *J*_m_ rates to drop on average to 40% of the basal *J*_m_ rate (Fig. 3C). This corresponds to 44% of *J*_m_ before the addition of glucose to cells cultivated with 11 m*M* glucose (denoted as *J^11^*_m0_; Fig. 3C). MnSOD and total SOD activities were constant (Supplementary Fig. S4A, B).

Isolated PIs kept in 5.5 m*M* glucose exhibited a similar decrease in *J*_m_ after glucose supplementation to 25 m*M* (Fig. 3E). For this assay, confocal monitoring was replaced with fluorimetry, while surveying fast-recorded MitoSOX Red spectra over the time course of the experiment (Supplementary Fig. S3H).

Next, we verified the MitoSOX Red confocal monitoring data by using a MitoB probe and liquid chromatography–mass spectrometry (LC-MS) quantification of its oxidized product MitoP (14, 36, 67, 68). Using an identical experimental set-up, but prolonged to 2 h after the addition of glucose, we clearly found less MitoP formed after high-glucose (25 m*M*) 2-h treatment when compared with 3 m*M* glucose. This was reflected by the lower MitoP/(MitoB+MitoP) ratios in INS-1E cells (Fig. 3F, left panel) and PIs (Fig. 3G, H). Again, similar data were obtained on NNT silencing (Supplementary Fig. S2Dd) and in PIs isolated from NNT-deficient C57BL6/J (Fig. 3G) and NNT-normal C57BL6/N mice (Fig. 3H), evidencing that the NNT absence in INS-1E cells and in C57BL6/J mice (58) does not affect the ROS decline on GSIS. This decline persisted also after 2 h of incubation with high glucose, when monitored with MitoSOX Red (Supplementary Fig. S5A, B). An entirely opposite response was found when Mito-paraquat was added to elevate oxidative status in the mitochondrial matrix of INS-1E cells (Fig. 3F, right panels). In conclusion, independent verification by LC-MS and MitoB confirmed diminished mitochondrial superoxide formation on GSIS.

**FIG. 4.**
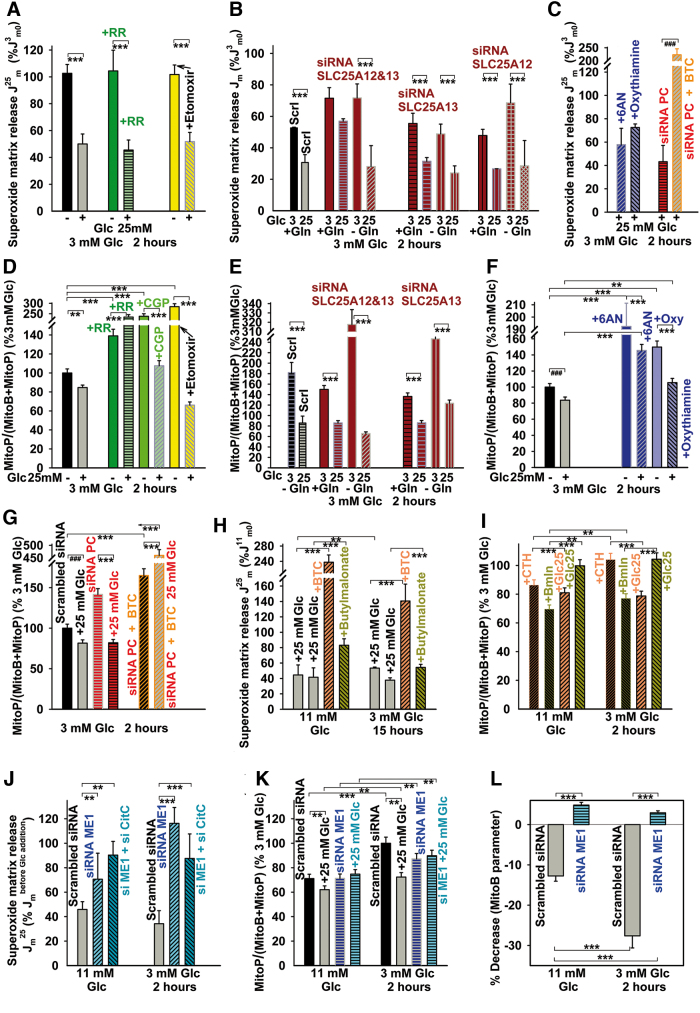
**MitoSOX Red and MitoB responses in the presence of various inhibitors of carriers and metabolism, on silencing ME1, pyruvate carboxylase, or aspartate/glutamate carriers of the malate/aspartate shuttle.** Mitochondrial matrix superoxide release rates *J*_m_ **(A–C, H, J)** and changes in 2 h-accumulation of H_2_O_2_/ROS **(D–G, I, K, L)** on transition between 3 and 25 m*M* glucose: **(A, D)** in the presence of RR (*green*) (7 μ*M*), etomoxir (100 μ*M*) (*yellow bars*), and CGP37157 (10 μ*M*) (*light green bar*); **(B, E)** on blockage of components of the malate/aspartate shuttle (*dark red bars*), that is, silencing of aspartate/glutamate antiporters SLC25A12/AGC1/aralar and SLC25A13/AGC2. **(C, F)** PPP blockage using 1 m*M* 6AN or 40 μ*M* oxythiamine (*blue bars*); **(C, G)** pyruvate carboxylase silencing (*red/orange bars*); for scrambled siRNA cf. **(J, K)**; **(H, I)** assays with CitC (10 m*M* BTC; *orange bars*) and 2OGC inhibitor (5 m*M n*-butylmalonate; *yellow-green bars*); and **(J–L)** assays on silencing cytosolic malic enzyme (“siRNA ME1”) (*dark blue* or *aquamarine bars*) alone or together with CitC (“si ME1+ si CitC”) as compared with scrambled siRNA (*gray bars*). *J*_m_ rates obtained in the presence of 25 m*M* glucose (*J*^25^_m_) were normalized either to rates obtained in 11 m*M* glucose before GSIS (*J*^11^_m0_), rates obtained in 3 m*M* glucose before GSIS (*J*^3^_m0_), or their respective initial glucose concentration before glucose addition (*J*_m_^before Glc addition^). ROS accumulation was normalized to 3 m*M* glucose. All preincubations in 3 m*M* glucose before the assay were performed for 2 h. ANOVA (*n* = 3–6): **p < 0.05; ***p < 0.001; Student's *t*-test: ^###^*p* < 0.001. 6AN, 6-aminonicotinamide; PPP, pentose-phosphate pathway; RR, ruthenium red. Color images are available online.

Glucose-induced decrease in *J*_m_ rates was also observed in the presence of etomoxir (Fig. 4A) (24), AOA (of a lower extent) (Supplementary Fig. S2Cc), and when the malate/aspartate shuttle (Fig. 1B) (42) should be eliminated by silencing the participating aspartate/glutamate antiporters SLC25A12/AGC1/aralar and SLC25A13/AGC2 or both, independently of glutamine presence (Fig. 4B). Glutamine itself decreased *J*_m_ rates, due to the accelerated respiration, and also due to the malate/aspartate shuttle blockage, which decreased *J*_m_ rates at 3 m*M* glucose down to 55–70% (Fig. 4B). Similarly, ruthenium red, a blocker of the Ca^2+^ uniporter (Fig. 4A), and PPP inhibitors (6-aminonicotinamide [6AN] and oxythiamine) (Fig. 4C) elicited a similar outcome.

Analogical patterns were obtained with MitoB (Fig. 4D–F), despite a massively higher H_2_O_2_ accumulation at 3 m*M* glucose with etomoxir, ruthenium red, CGP37157, at inhibited PPP, and when omitting glutamine. The declines with etomoxir, at inhibited PPP, and without glutamine were high (Fig. 4D–F). Due to the inhibition of respiration/OXPHOS, the observed decrease in accumulated ROS (and also the onset of oxidative stress with ruthenium red) was relatively nonspecific on blockage of the Ca^2+^/Na^+^ efflux with CGP37157 (Fig. 4D). The discrepancy of increasing accumulated ROS *versus* the MitoSOX assay results could stem from such nonspecific effects. Nevertheless, there was a decrease in the 2 h-accumulated ROS with AOA and when the malate/aspartate shuttle (42) was eliminated by silencing SLC25A12/AGC1/aralar or SLC25A13/AGC2 and both (Fig. 4E and Supplementary Fig. S2Cb, Cd).

### Mitochondrial matrix H_2_O_2_ release slows down in INS-1E cells on glucose addition

Using mito-HyPer, a H_2_O_2_ selective protein-based probe targeted to the mitochondrial matrix (4), we monitored time courses of H_2_O_2_ release into the mitochondrial matrix under identical conditions to the MitoSOX Red monitoring. As expected, the corresponding *J*_m_^H2O2^ rates decreased on GSIS, hence independently confirming MitoSOX Red results by a third methodical approach.

To eliminate any possible dependency of mito-HyPer on pH, the H_2_O_2_-insensitive probe mito-SypHer was used in parallel (15). Any interfering contribution of pH was compensated for by subtracting mito-SypHer from the mito-HyPer fluorescence signal. The signal was calculated as the integral of fluorescence in the regions of interests (ROI) per unit area within the equal area of the mitochondrial network for each of two excitations at 488 and 405 nm. The resulting differential fluorescence data (Δ*F*) were used to calculate the *R*(*H_2_O_2_*) ratios of corrected mito-HyPer emission excited at 488 *versus* 405 nm (*R*(*H_2_O_2_*) = Δ*F ^H^*_485_**/**Δ*F ^H^*_405_) (Fig. 3I). Despite having an approximate calibration, that is, since not all of the externally added H_2_O_2_ penetrates into the mitochondrial matrix and influences the probes localized there, we expressed the slopes of the derived dependencies as upper limits to rates of H_2_O_2_ production *J*_m_^H2O2^ in nmol·s^−1^.

The main features of the resulting H_2_O_2_ monitoring (Fig. 3I–N) were as follows: In the presence of rotenone, a permanently increased *R*(*H_2_O_2_*) was observed, giving a certain positive *J*_m_^H2O2^ rate and reflecting the H_2_O_2_ release into the mitochondrial matrix (Fig. 3K, L). In contrast, relatively negative *J*_m_^H2O2^ rates were obtained for INS-1E cells in 3 (Fig. 3I) and 11 m*M* glucose (Fig. 3J), thus indicating either an actual H_2_O_2_ efflux from the mitochondrial matrix or alternatively a decrease in H_2_O_2_ formation (the two options being indistinguishable). The subsequent glucose addition (25 m*M*) led to a further decrease in H_2_O_2_ release into the mitochondrial matrix (or an increase in H_2_O_2_ efflux, or alternatively both). Consequently, higher negative *J*_m_^H2O2^ rates were apparent at 25 m*M* glucose (Fig. 3M, N). This qualitatively confirms the results obtained by using MitoSOX Red superoxide monitoring (Fig. 3C) or MitoB (Fig. 3F).

Validation of positive/negative *J*_m_^H2O2^ rate transitions was done by using mitochondria-specific antioxidants, which prevent rotenone-induced superoxide generation, and hence H_2_O_2_ formation in mitochondria *in vivo*. Mitochondria-targeted antioxidants, SkQ1 and suppressor of Complex I site Q electron leak (S1QEL) changed the positive *J*_m_^H2O2^ rates into negative ones. These results reflect the powerful antioxidant action of these agents within the mitochondrial matrix (Fig. 3K, L).

### Mitochondrial matrix superoxide release at blocked redox shuttles

Three redox shuttles in pancreatic β cells should hypothetically facilitate GSIS by exporting reducing equivalents from the mitochondrial matrix into the cytosol (34), where ME1 or IDH1 produces NADPH (Fig. 1A) (35). It should be noted that two PPP enzymes also form NADPH. Since such export of reducing equivalents should affect matrix redox homeostasis, we evaluated changes in the matrix superoxide release *J*_m_ (Fig. 4H) and 2-h accumulation of H_2_O_2_/ROS in INS-1E cells (Fig. 4I) and PIs (Supplementary Fig. S6) while blocking the redox shuttles by inhibiting citrate transport with BTC (35) (Fig. 4H and Supplementary Figs. S1Ca and S2Dc) and 4-chloro-3-[[(3-nitrophenyl)amino]sulfonyl]-benzoic acid (CTH) (Fig. 4I); and also inhibiting 2OGC by *n*-butylmalonate (Fig. 4H, I and Supplementary Fig. S1Da). In the presence of mitochondrial metabolite carrier inhibitors, *J*_m_ rates increased (Fig. 4H) and H_2_O_2_/ROS accumulation did not significantly change or, in some instances, even increased on glucose addition (Fig. 4I and Supplementary Fig. S6).

INS-1E cells with silenced ME1 (or ME1 plus CitC) exhibited much higher *J*_m_ rates at 3 or 11 m*M* glucose, which were less retarded or were increased on glucose addition (25 m*M*) when compared with samples with scrambled small interfering RNA (siRNA) (Fig. 4J and Supplementary Fig. S1Ab). This was confirmed by MitoB (Fig. 4K, L). Glucose-induced decreases in 2-h accumulation of H_2_O_2_/ROS ceased with ME1 silencing (Fig. 4K, L). Thus, the redox shuttle turnover was retarded, due to the blockage of key components by silencing (90% of ME1 transcript vanished, and 60% of CitC transcript when silenced simultaneously, or 90% when alone; Supplementary Fig. S1Ab). The slower metabolic turnover resulted in higher superoxide release to the mitochondrial matrix. Similarly, the silencing of IDH2 allowed by ∼20% lower decline of *J*_m_ rates on transition from 3 to 25 m*M* glucose (Supplementary Fig. S2Eb, Ec) reflected the shutdown of only one among the three shuttles (Supplementary Fig. S2Ea). In MitoB response, analogical decline vanished with IDH2 silencing (Supplementary Fig. S2Ed).

In INS-1E cells with silenced pyruvate carboxylase, the decline in *J*_m_ rates was unchanged, but with the simultaneous inhibition of CitC using BTC, the mitochondrial matrix superoxide release doubled (Fig. 4C and Supplementary Fig. S1Ba, Bb). Also the decline in accumulated H_2_O_2_/ROS was more pronounced, but the further addition of BTC again led to a high oxidative stress at low and high glucose, which was much higher for the latter (Fig. 4G).

### Mitochondrial redox shuttles are responsible for the increase in cytosolic NADPH and matrix NAD^+^ on GSIS

**FIG. 5.**
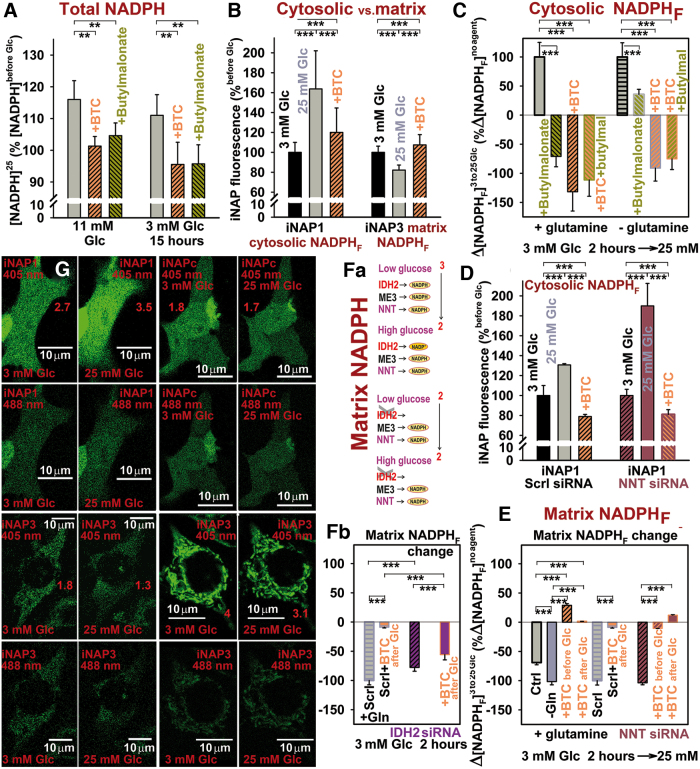
**Cytosolic NADPH elevation on GSIS. (A)** Total cell NADPH was assayed by the BioVision kit, ANOVA (*n* = 3–6) **p < 0.05; **(B–D, G)** cytosolic NADPH elevations, or **(E–G)** NADPH declines within the mitochondrial matrix, assayed by the iNAP1 or iNAP3 fluorescence probes, respectively, in INS-1E cells (preincubated with 3 m*M* glucose for 2 h) on glucose addition to the final concentration of 25 m*M*; in the absence or presence of CitC inhibitor (10 m*M* BTC, *orange bars*) or 2OGC inhibitor (5 m*M n*-butylmalonate, *yellow-green bars*) or both; or in NNT-silenced **(***brown bars*, **D, E)** or IDH2-silenced cells **(***purple bars*, **Fb)**. Inhibitors were added after the glucose addition **(B–D)**. Images in **(G)** show representative cells transfected with iNAP1 and iNAP3, respectively, or nonresponding iNAPc observed by confocal microscopy at 405 and 488 nm excitations. Emission ratios 405/488 are indicated by numbers. **(Fa)** The most probable enzyme contribution to the mitochondrial matrix NADPH pool, under the assumption that NNT always acts in its forward mode (combinations of all NNT possible modes see Supplementary Fig. S2Ef) and of equal enzyme contribution to the NADPH pool. It is deduced that IDH2 deletion should lead to a large elimination of the matrix NADPH_F_ drop on GSIS. This trend was observed only for IDH2-silenced cells with BTC. ANOVA for **(B, C)** *n* = 40–60 image spots or **(Fb, B)** *n* = 35–55; *n* = 85 for controls: ***p < 0.001. Color images are available online.

An enzyme-based assay of the total cell NADPH confirmed an increase (>10%) in total NADPH on glucose being set to 25 m*M* in INS-1E cells (preincubated with 11 or 3 m*M* glucose; Fig. 5A). This increase was prevented by CitC inhibition with BTC (Fig. 5A) or CTH; and by the 2OGC inhibitor *n*-butylmalonate (Fig. 5A). Parallel confocal fluorescence monitoring of free cytosolic NADPH_F_, using the iNAP1 fluorescence probe (Fig. 5B–D; as compared with the insensitive iNAPc probe, see Fig. 5G), indicated an [NADPH_F_]_c_ increase on transition from 3 to 25 m*M* glucose. These changes were prevented or, in some instances, reversed by BTC and *n*-butylmalonate (Fig. 5B–D and Supplementary Fig. S7). Thus, the active redox shuttles involving CitC and 2OGC provide the export of reducing equivalents from the mitochondrial matrix (Fig. 1A). The data were similar on NNT silencing (Fig. 5D), confirming that the redox shuttles act upstream of NNT. IDH2 silencing insignificantly inhibited the [NADPH_F_]_c_ elevation (Supplementary Fig. S2Ee), since the two remaining shuttles should be still operating, unlike with BTC in IDH2-silenced cells, which left only one, thus preventing the cytosolic [NADPH_F_]_c_ elevations.

Simultaneously, the matrix-addressed iNAP3 indicated a decrease in mitochondrial [NADPH_F_]_m_ on GSIS (Fig. 5E–G). The matrix NADPH/NADP^+^ homeostasis stems mainly from the complex contribution of NNT, IDH2, and the NADP^+^-dependent malic enzyme ME3 (Supplementary Fig. S2Da, Ea, Ef). However, the iNAP3-monitored drop in [NADPH_F_]_m_ was not affected on NNT silencing (Fig. 5E). Figure 5Fa and the simplified S2Ef schematics explain why the matrix [NADPH_F_]_m_ should not decline on IDH2 silencing. However, we observed only a lesser decline (high decline with BTC; Fig. 5Fb), resulting from an unequal contribution of the considered enzymes.

### Accumulation of selected metabolites and citrate/isocitrate export on GSIS

**FIG. 6.**
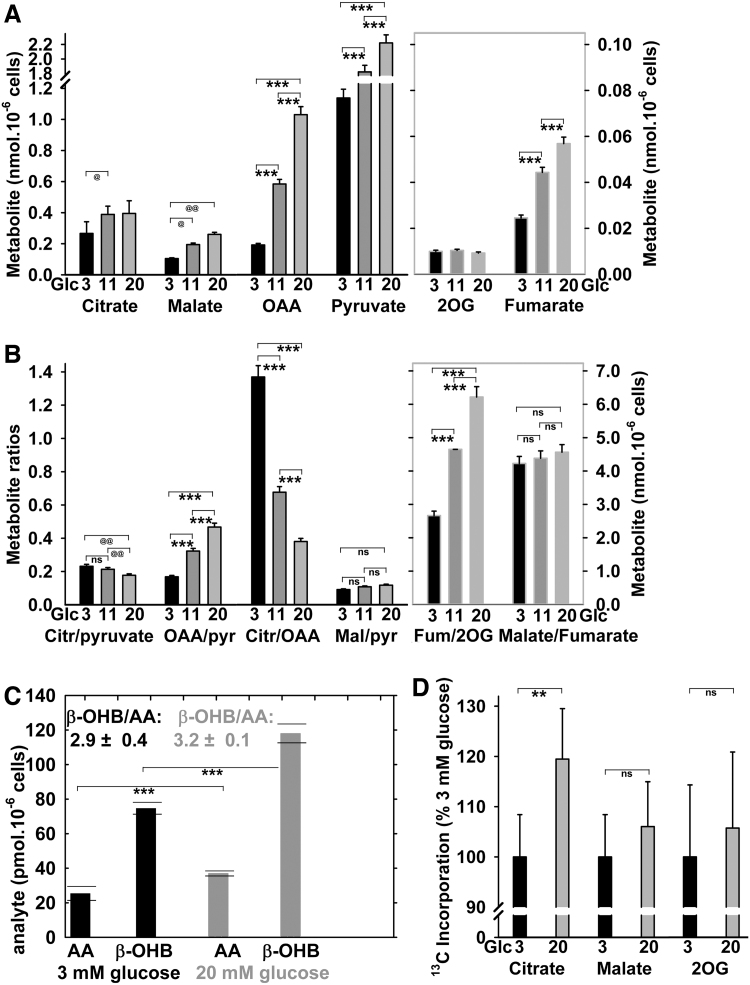
**Targeted metabolomics of the Krebs cycle intermediates, acetoacetate, and β-OHB and evidence for the isocitrate/pyruvate redox shuttle.** Total cellular metabolite levels **(A)**, their selected ratios **(B)**, and AA and β-OHB and their ratios as indicated **(C)** were quantified in INS-1E cells preincubated with 3 m*M* glucose for 1 h; then, glucose was raised by zero, 8, and 17 m*M* surplus to reach a final concentration of 3 m*M* (*black bars*), 11 m*M* (*dark gray bars*), and 20 m*M* (*gray bars*) glucose and subsequently incubated for another 30 min. ANOVA for **(A, B)** yielded (*n* = 5) ***p < 0.001; whereas Student's *t*-test for **(A, B)** yielded (*n* = 5): ^@@^*p* < 0.05; ^@^*p* < 0.1; and for **(C)** (all estimates from two independent experiments are shown with averages and SDs); *p* < 0.001 for all combinations between the two compounds. The difference between the β-OHB/AA ratios was not significant. Notably, the significant β-OHB increase suggests also the increase in mitochondrial matrix NAD^+^, since β-OHB dehydrogenase, which exists only in the mitochondrial matrix, produces β-OHB from AA at the expense of NADH, thus forming NAD^+^ (46). The fact that AA does not proportionally decrease reflects other reactions (43, 46) and penetration of AA into the cytosol during the sample preparation (Supplementary Fig. S2Fa). **(D)** ^13^C incorporation from 1-^13^C-l-glutamine into citrate, malate, and 2OG is expressed for normalized data for 25 m*M* glucose (2-h incubations) in relation to average values obtained after a 2-h incubation with 3 m*M* glucose. Data were first calculated in % of ^13^C accumulated amounts *versus* total (^13^C+^12^C) amount of a given compound when accounted for the natural ^13^C content. Evidence for the isocitrate/pyruvate redox shuttle is suggested by the existence of the ^13^C-accumulation, as such. This is because the ^13^C-accumulation into citrate from 1-^13^C-glutamine cannot exist on the forward Krebs cycle, since ^13^C-CO_2_ is formed and eliminated from the sample; hence any ^13^C-labeled citrate or malate molecule (when subtracting those with naturally occurring ^13^C) must originate from the reverse Krebs cycle direction, given by the IDH2-mediated NADPH-driven reductive carboxylation of 2OG. ANOVA for **(D)** (*n* = 6): ^**^p < 0.05. Also, insignificantly (“ns”) increased ^13^C incorporation into malate and 2OG is indicated. β-OHB, β-hydroxybutyrate; AA, acetoacetate; ns, nonsignificant; SDs, standard deviations.

Among Krebs cycle metabolites, citrate, malate, fumarate, and oxaloacetate (OAA) were increased at 20 *versus* 3 m*M* glucose (Fig. 6A). Metabolite accumulation reflects either an increase in their supply or a decrease in their utilization (61, 62). An unchanged malate/fumarate ratio could indicate proportional increases in respiration and turnover of this Krebs cycle segment (Fig. 6B). The decreasing citrate/pyruvate ratio reflects more rapid pyruvate utilization with 20 m*M* glucose. The elevated OAA/pyruvate ratio at the decreasing citrate/OAA ratio supports the increasing pyruvate carboxylase reaction and citrate synthase reaction on GSIS. Also, 2-oxoglutarate (2OG) increased on GSIS, despite being present at lower concentrations due to a high turnover. Estimation of β-hydroxybutyrate (β-OHB) to acetoacetate ratio indicated its insignificant small rise on GSIS (Fig. 6C). The observed β-OHB elevation supports the matrix NAD^+^ increase (Supplementary Fig. S2Fa) (43, 46), since the rat β-OHB dehydrogenase exists only in the matrix.

The existence of ^13^C-incorporation, from 1-^13^C-glutamine into ^13^C-citrate, ^13^C-2OG, or ^13^C-malate, evidenced the operation of the isocitrate/pyruvate shuttle, specifically the reductive carboxylation reaction of IDH2 (Fig. 6D) (59). Despite quantifications of ^13^C-labeled metabolites in INS-1E cells after a 2-h incubation having lower resolution *versus* those performed after 6 h (59), we detected about a 1.2-fold rise of ^13^C-incorporation into ^13^C-citrate and a 1.1-fold rise of ^13^C-malate from 1-^13^C-glutamine, when comparing 3 *versus* 20 m*M* glucose. Here, the increased ^13^C-incorporation represents a higher reaction turnover, since initially, only the natural ^13^C-content exists in analyzed metabolites. The latter was actually subtracted from the measured data of Figure 6D.

### Two-channel fluorescence lifetime imaging microscopy-assessed NADPH and NADH or NAD^+^ responses to glucose

**FIG. 7.**
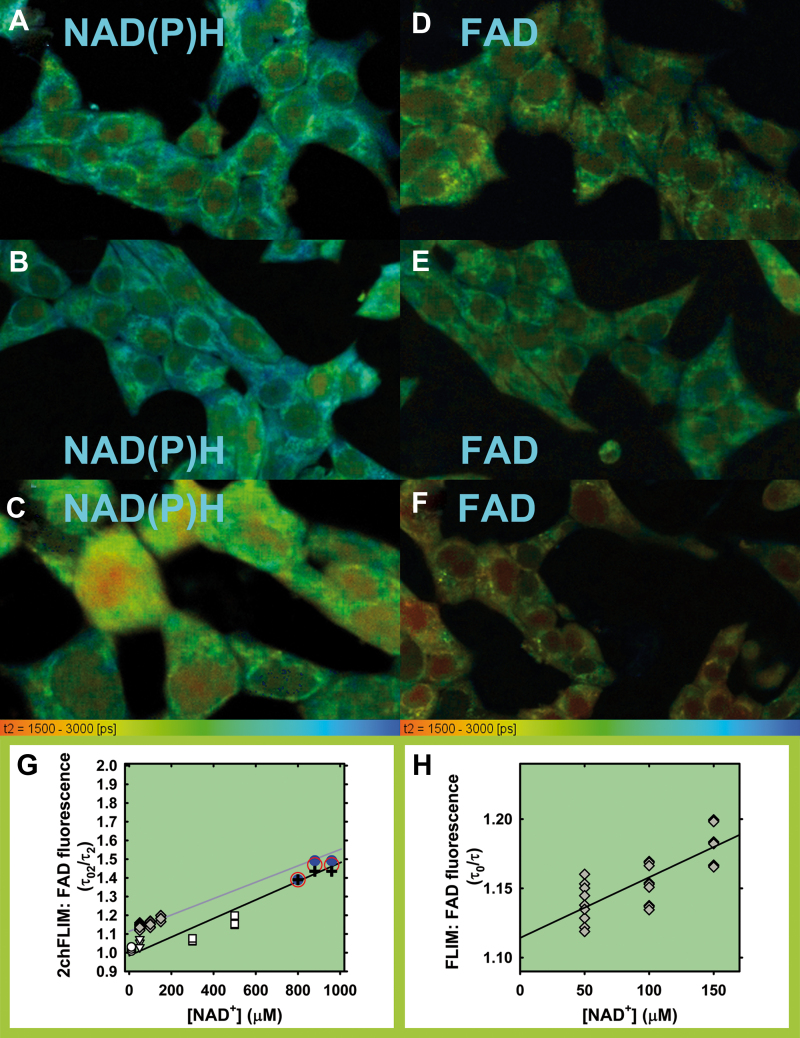
**2chFLIM images of τ_2_ mode for bound NAD(P)H and free FAD signals and approximate calibration. (A, D)** INS-1E cells cultured in medium with 11 m*M* glucose (“Glc11”); **(B, E)** after glucose addition to reach a final concentration of 25 m*M* (“Glc25”); **(C, F)** as **(B, E)** with 10 m*M* BTC. **(G, H)** Approximate calibration. Decreases in single-component resolved FAD emission decay **(**details in **H)**, expressed by the linearized Stern-Volmer relationship (eq. {12}) when 1 m*M* FAD in glycerol was titrated by using 50 μ*M* NAD^+^ aliquots (*gray data* and *gray fit*). A single decay component τ_0_ was estimated in the absence of quencher (NAD^+^), whereas component τ corresponded to the studied NAD^+^ additions **(H)**. Data in **(G)** (except of *gray symbols*) show the τ0_2_^FAD^ from the FAD channel of 2chFLIM relatively to the decreasing τ_2_^FAD^. INS-1E cells were permeabilized by using digitonin (0.5–1 μg), whereas we measured and analyzed the FAD emission of either (i) natural cytosolic FAD content (*circles*, *triangles*); or (ii) added FAD (10 or 100 μ*M*; *squares*). Parameter τ0_2_^FAD^ was estimated first, before addition of NAD^+^ aliquots. *Blue* or *red circles* and *black crosses*: experimental data from Figure 8F: *Blue circles*—maximum acquired shift in τ_2_^FAD^ on GSIS; *red circles*—an average shift in τ_2_^FAD^, both from the initial 11 m*M* glucose; *black crosses*—an average shift in τ_2_^FAD^ from the initial 3 m*M* glucose. Shifts in x-axis show 10% and 20% elevations in NAD^+^ concentration. The data approximately fit to the Stern-Volmer relationship for dynamic quenching (eq. {12}), to which also our experimental τ_2_^FAD^ data (lifetimes for free FAD) from Figure 8F fit well under the assumption that mitochondrial matrix NAD^+^ is around 800 μ*M* and was 10% or 20% elevated on GSIS. Note also that if this NAD^+^ was overestimated and may be lower around 500 μ*M*, a slightly different Stern-Volmer relationship (eq. {12}) still would be valid. 2chFLIM, two-channel fluorescence lifetime imaging microscopy. Color images are available online.

**FIG. 8.**
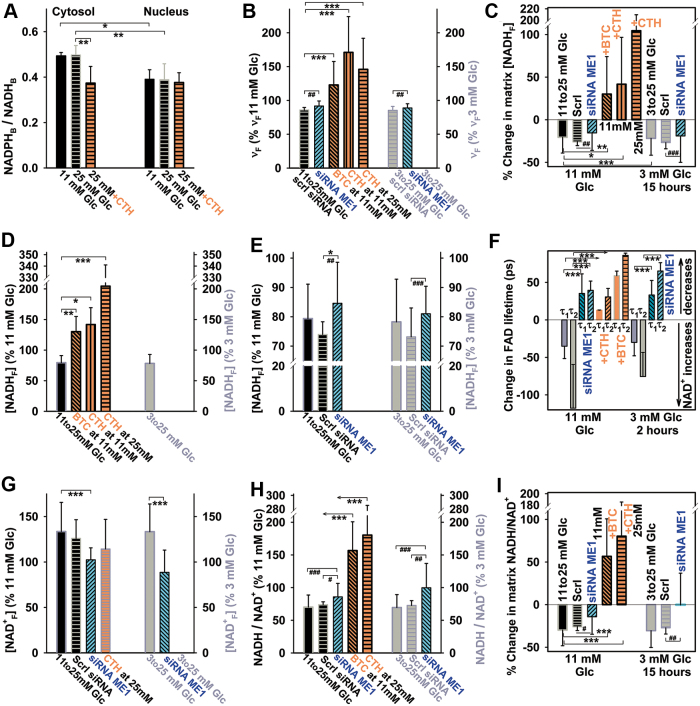
**2chFLIM-derived changes in mitochondrial matrix NADH, NAD^+^, and NADH/NAD^+^ ratio compared with cytosolic bound NADPH/NADH ratios on GSIS. (A)** Cytosolic and nuclear-bound NADPH/NADH ratios derived from 2chFLIM according to Blacker *et al.* (6) in extramitochondrial ROI and nuclear ROI is shown for INS-1E cells. **(B–I)** Relative changes in mitochondrial matrix on glucose elevation to 25 m*M* for coefficient ν_F_ as calculated by using eq. {11} **(B)**; estimated free NADH (unbound; NADH_F_) **(C–E)**; free NAD^+^ (derived from 2chFLIM on the basis of FAD signal quenching by NAD^+^, using eq. {13}); and approximated changes in substrate pressure *S*—where *S* = NADH_F_/NAD^+^_F_ and changes are expressed as *S*(*2*)/*S*(*1*) in percentages, where *S*(*1*) denotes the substrate pressure before and *S*(*2*) after GSIS for free compounds, using eq. {15}. BTC, 10 m*M*, CTH, 0.5 m*M*. Data were calculated by using the integral parameters from the mitochondrial network ROI **(**except for **A)** and expressed as averages ± SD of analyzed *N* biological replicates (2chFLIM NAD(P)H autofluorescence images), each typically containing 80–100 cells, while having *n* estimations in each. ANOVA: ***p < 0.001; **p < 0.05; *p < 0.1; Student's *t*-test: ^###^*p* < 0.001; ^##^*p* < 0.01; ^#^*p* < 0.1. *N*/*n* for INS-1E cells preincubated with 11 m*M* glucose was 13/31 (7/17 siRNA ME1; 7/21 with CTH; 3/9 with BTC); whereas with 3 m*M* glucose *N*/*n* was 16/41 (6/16 siRNA ME1). CTH, 4-chloro-3-[[(3-nitrophenyl) amino] sulfonyl]-benzoic acid. Color images are available online.

We also attempted to evaluate changes in the mitochondrial matrix NADH or NAD^+^ and trends in changes of NADH/NAD^+^ ratios by using our novel two-channel fluorescence lifetime imaging microscopy (2chFLIM) method (Figs. 7 and 8), which is able to assess these changes without the need of precise quantifications of separate NADH and NAD^+^ concentrations. In this way, we independently tested our hypothesis that the mitochondrial matrix NADH/NAD^+^ ratio decreases when the redox shuttles (Fig. 1A) are active (Fig. 8B–E). At first, using the quantification developed by Duchen and colleagues (6), we found that on transitions from 3 or 11 to 25 m*M* glucose, the ratios of bound species NADPH_B_/NADH_B_ derived from the 2chFLIM data within chosen ROI did not significantly change in the matrix (Supplementary Fig. S8) and slightly increased in the cytosol (Fig. 8A). Unlike in the nucleus, the cytosolic NADPH_B_/NADH_B_ ratio decreased on the inhibition of CitC (Fig. 8A).

We then extended Duchen's method to estimate the concentration of free mitochondrial NADH ([NADH]_F_) based on the Scatchard equation and the assumption of the predominant participation of Complex I. Consequently, [NADH]_F_ was approximated according to eq. {10} from the corresponding decay coefficient ν_F_ (Fig. 8B) and the initial NAD(P)H autofluorescence intensity, whereas changes after glucose addition were calculated according to eq. {11} (Fig. 8C–E). These estimates showed that [NADH]_F_ decreased on average by 20% on GSIS (Fig. 8C–E). The coefficient ν_F_ itself declined on average by 12% (Fig. 8B). The decrease ceased or changed to an increase in [NADH]_F_ when the citrate export was inhibited with BTC or CTH (Fig. 8C, D) and when ME1 was silenced (Fig. 8E), that is, when blocking at least two redox shuttles.

A faster decay of FAD autofluorescence on GSIS in INS-1E cells was indicated by shorter lifetimes of both bound FAD (a short τ_1_^FAD^ = τ^FAD^_B_) and free FAD (a longer τ_2_^FAD^ = τ^FAD^_F_) (Fig. 8F). This could indicate the existence of the augmented quenching of FAD by NAD^+^, thus indicating an NAD^+^ increase. Assuming NAD^+^ in 500–800 μ*M*, the obtained τ_2_^FAD^ values fit into the 2chFLIM calibration performed in permeabilized cells (Fig. 7G, H). Trends in [NAD^+^]_F_ changes are summarized in Figure 8F and G. In contrast, when the citrate export was inhibited with BTC (CTH) or ME1 was silenced, both lifetimes of FAD emission either did not significantly change or even increased (Fig. 8F). The latter indicates a partial loss of quenching and could be ascribed to lower [NAD^+^]_F_ (Fig. 8G). Otherwise, observations of a rise in mitochondrial matrix NAD^+^ on GSIS further support our Hypothesis I, that the active redox shuttles effectively increase mitochondrial NAD^+^, not allowing the extensive IDH3 and MDH forward reactions. This leads to the accumulation of matrix NAD^+^. Obviously, the inhibition of citrate/isocitrate export re-accelerates IDH3, and hence the NAD^+^ increase ceases. Similarly, the pyruvate/malate and the pyruvate/citrate redox shuttle (requiring also CitC) are dependent on the ME1 reaction, as the ME1 silencing causes the disappearance of the observed changes on GSIS.

Despite several simplifications introduced into our calculations of separate concentrations of nicotinamide nucleotides in the mitochondrial matrix and cytosol, we obtained estimates of NADH/NAD^+^ ratios in the matrix as decreasing on GSIS (Fig. 8H, I). Both ways, division of the estimated changes in [NADH]_F_ by those in [NAD^+^]_F_ and the approximation using eq. {15} gave similar results. On CitC inhibition with BTC or CTH and silencing of ME1, the mitochondrial matrix NADH/NAD^+^ ratios either remained unchanged or increased (Fig. 8H, I).

### Origin of mitochondrial matrix superoxide release

**FIG. 9.**
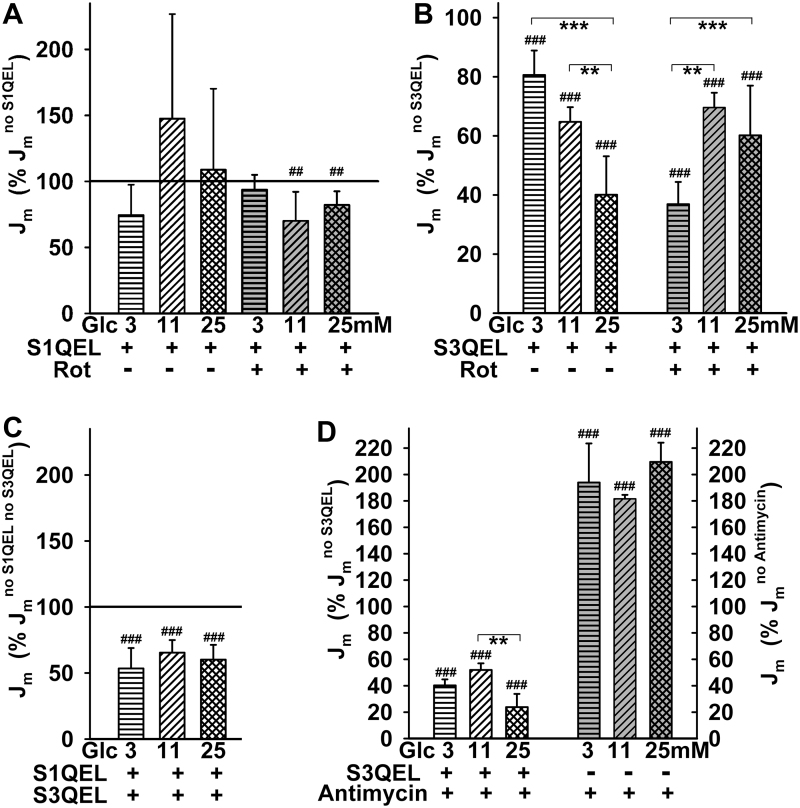
**Effects of suppressors of electron leak at specific sites. (A)** Effect of S1QEL—Mitochondrial matrix superoxide release *J*_m_ rates were normalized to those without S1QEL at varying glucose concentrations in the absence and presence of rotenone as indicated. **(B)** Antioxidant capacity of S3QEL—*J*_m_ rates were normalized to those without S3QEL in the absence or presence of rotenone. **(C)** Simultaneous effects of S1QEL plus S3QEL at 11 m*M* glucose. *J*_m_ rates were normalized to those without both agents. (**D**) Antioxidant effect of S3QEL towards Antimycin-induced superoxide production. S1QEL and S3QEL, 10 μ*M*; rotenone, 20 μ*M*. ANOVA (*n* = 4–12): ^##^*p* < 0.01; ^###^*p* < 0.001; when normalized to 100%. **p < 0.01; ***p < 0.001; when compared among samples. S3QEL, suppressor of complex 3 site Q electron leak.

To ascribe the fractions of mitochondrial matrix superoxide release in INS-1E cells to specific sites of superoxide formation, we employed suppressors of electron leak (Fig. 9A–D), developed by Brand *et al.* (9). S1QEL exhibited virtually no antioxidant effect at 3 m*M* glucose and acted as a pro-oxidant at 11 and 25 m*M* glucose (Fig. 9A). At the same time, S1QEL exerted a weak antioxidant effect against rotenone-induced superoxide production at 11 and 25 m*M* glucose (Fig. 9A) (29). These results demonstrate that both rotenone and S1QEL interfere within the I_Q_ site of Complex I in the vicinity of the ubiquinone binding site (29).

In contrast, the suppressor of Complex III site Q electron leak (S3QEL) exhibited an antioxidant effect that increased with increasing glucose, diminishing *J*_m_ rates down to 81%, 65%, and 40% at 3, 11, and 25 m*M* glucose, respectively (Fig. 9B). Indirectly, these results indicate that the remaining portion of the superoxide matrix release, which also includes the contribution from the I_F_ site, decreases with increasing glucose on GSIS. This perfectly correlates with the decreasing substrate pressure *S*. Thus, on GSIS with maximum glucose, at least 60% of the superoxide released into the mitochondrial matrix originates from the Complex III outer Q site III_Qo_ (7, 8, 29). In contrast, at 3 m*M* glucose, the contribution of site III_Qo_ is only 20% and the remaining superoxide is produced predominantly from the I_F_ site due to the high substrate pressure.

Unexpectedly, S3QEL also exhibited an antioxidant effect against rotenone, being the most efficient at 3 m*M* glucose (Fig. 9B). The site specificity for Complex III was confirmed by the observation of the nearly complete S3QEL-mediated suppression of superoxide release induced by antimycin A (Fig. 9D). Further, addition of S1QEL together with S3QEL did not further decrease the *J*_m_ rates, which remained at 53%, 65%, and 60% at 3, 11, and 25 m*M* glucose, respectively (Fig. 9C). Comparing this with the effects of S3QEL alone, the slight pro-oxidant action of S1QEL still contributes to the composite effect.

## Discussion

**FIG. 10.**
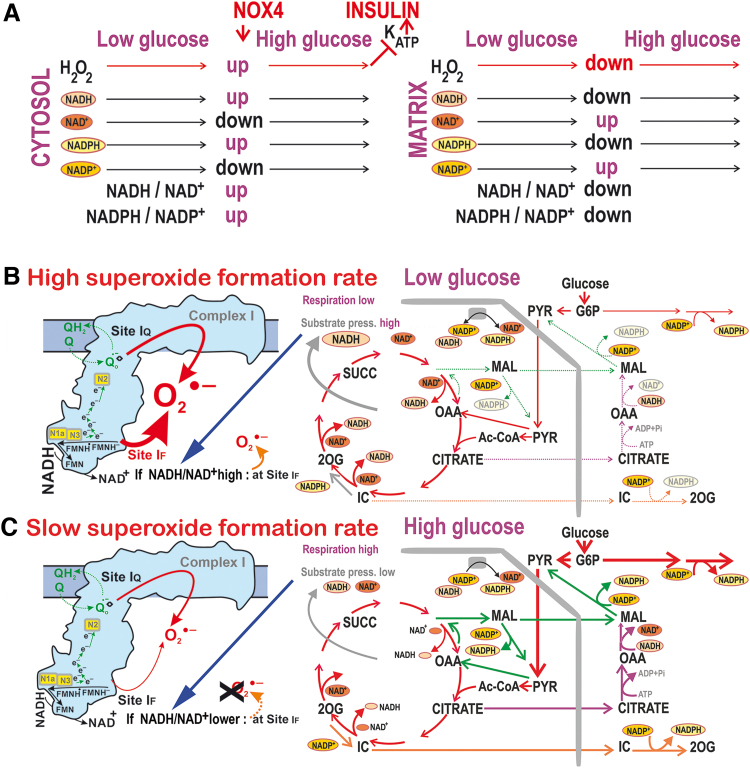
**Major metabolic fluxes and redox shuttles contributing to diminished mitochondrial superoxide generation in pancreatic β cells on GSIS. (A)** Overview of changes on GSIS in the cytosol and mitochondrial matrix; **(B)** Cells at low glucose; **(C)** cells on glucose intake: Higher rates of metabolic fluxes are depicted by thick arrows, whereas lower rates are shown by dotted arrows and decreasing products are depicted in *gray fonts* or *symbols*. The color coding for shuttles and abbreviations are the same as in Figure 1. Similarly, higher superoxide formation rate is indicated with *thick arrows*, whereas slow rate is depicted with *thin arrows*. Color images are available online.

We described a decline in mitochondrial superoxide formation during glucose-induced insulin secretion in rat pancreatic β cells (INS-1E cells) and PIs. We not only confirmed the recently reported glucose-induced reduction in oxidation of mitochondrial matrix-targeted roGFP2-Orp1 (15) but also elucidated the mechanism for the decreased pro-oxidant state, established in the mitochondrial matrix on GSIS. A concomitant drop in the matrix NADH_F_/NAD^+^_F_ and NADPH_F_/NADP^+^_F_ was indicated, in contrast to elevations in the cytosolic NADPH_F_ (Fig. 10A). Since the decreased matrix NADPH_F_/NADP^+^_F_ ratio may cause equivalent exhaustion of matrix reduced glutathione (GSH), we may conclude that the acute pro-oxidant state decrease in mitochondria on GSIS is established at the expense of susceptibility to the oxidative stress.

The acute phenomenon results from the finely tuned bioenergetics of the OXPHOS metabolism of glucose, switching toward increasing respiratory chain substrates in pancreatic β cell mitochondria and increasing the activity of three mitochondrial redox shuttles that subsequently export reducing equivalents to the cytosol (Fig. 10B, C). The resulting elevated cytosolic NADPH_F_ facilitates insulin secretion (34). Recently, we explained how, when revealing the existence of the essential NOX4-mediated cytosolic redox signaling, which together with ATP elevations fundamentally determines insulin exocytosis (48). A portion of NADPH_F_ required for NOX4 reaction comes from the redox shuttles described here. Other substantial NADPH_F_ supply for NOX4 is produced by two of the PPP enzymes on GSIS.

Since β cells lack a significant lactate dehydrogenase activity and functional pyruvate dehydrogenase kinases, nearly 100% of pyruvate originating from glycolysis is utilized by the Krebs cycle (2, 10, 52, 55) to sustain OXPHOS (Figs. 1A, B and 10B, C). Pyruvate is about equally metabolized by PDH and pyruvate carboxylase (52). The PDH enables an increase in the Krebs cycle rate, followed by the increased respiration (60) and OXPHOS, thus increasing ATP, which subsequently contributes to K_ATP_ closing and GSIS.

If only PDH was functional and without operating redox shuttles, the resulting incremental increase in NADH and concomitant elevation of respiration would cause a higher extent of proton pumping and establish a high protonmotive force Δ*p* (including higher Δ*Ψ*_m_). The concomitantly increased ATP synthesis would transport more protons back through the ATP-synthase. The resulting Δ*p* would thus be established as somewhat smaller than without this proton backflow. As a result, also mitochondrial superoxide formation would decrease (Hypothesis II). This component is a basic one, fundamentally contributing to the mitochondrial reductive state.

However, with the additional pyruvate carboxylase reaction and concomitant malate efflux from the matrix, enabled by 2OGC, the generated OAA can either increase the turnover of the Krebs cycle or be converted by the reverse MDH reaction and thus initiate the pyruvate/malate shuttle (Figs. 1A and 10B, C). MDH then produces less NADH than would be produced without the redox shuttle. Its operation was proven here by the effects of ME1 and pyruvate carboxylase silencing and inhibitors of the respective carriers.

Crucially, this lower matrix NADH availability is also given by the lower IDH3-mediated NADH formation due to the effective citrate and isocitrate efflux within the pyruvate/citrate and pyruvate/isocitrate shuttles, respectively. Their operation provides much lower matrix NADH/NAD^+^ ratios than in their absence. Matrix NADH in μ*M* but NAD^+^ of >500 μ*M* seems to be typical (12). Indeed, functionality of the pyruvate/isocitrate shuttle was independently supported by ^13^C-incorporation experiments (Fig. 6C), unequivocally determining the counter-Krebs cycle direction of isocitrate flux (59) and its increase at high glucose.

Under the hypothetical maximum substrate elevation without redox shuttles, one would have anticipated elevated superoxide formation due to the expected higher matrix NADH/NAD^+^ ratios. Such a high matrix substrate pressure *S* (NADH_F_/NAD^+^_F_) would cause higher superoxide formation at site I_F_ in the vicinity of the flavin binding site of Complex I (8, 50). The only situations that followed the most simple expectation of an increased superoxide formation with increased substrates occurred (i) in media completely depleted of glucose and pyruvate-free on glucose addition (Fig. 3A) or (ii) in cells with an inhibited citrate or malate export; or with two redox shuttles blocked by ME1 silencing, partly with pyruvate/isocitrate shuttle blocked by IDH2 silencing, or completely with simultaneous silencing of pyruvate carboxylase and inhibited citrate export; that is, again with inhibited redox shuttles. Otherwise, with initial 3 and 11 m*M* glucose, the sudden glucose intake slows down superoxide formation released to the mitochondrial matrix (Fig. 3A–E).

The three active redox shuttles allow elevations of cytosolic NADPH_F_ on GSIS (34, 35) (Figs. 5B–D and 8A) at the expense of the diminished mitochondrial matrix NADH_F_ and a concomitant NAD^+^_F_ increase. Its existence was also supported by a 3.2-fold increase in β-OHB (Fig. 6C and Supplementary Fig. S2Fa). Consequently, the resulting matrix substrate pressure (NADH_F_/NAD^+^_F_) is also diminished (Fig. 8H, I). When these shuttles are shut down by combinations of the metabolite carrier inhibitors with silencing of ME1, IDH2, or pyruvate carboxylase, the substrate pressure NADH_F_/NAD^+^_F_ does not decrease but instead increases (Fig. 8H, I). Thus, an elevated matrix superoxide release stems from the inhibited redox shuttles (Fig. 3A–D). Concomitantly, elevation of the cytosolic NADPH_F_ at high glucose vanishes with the blocked carriers and/or shuttles (Figs. 5A–D and 8A and Supplementary Fig. S7). The maintenance of NAD^+^ may also maintain the ongoing Sirtuin-3-mediated de-acetylation of proteins and factors that are crucial for OXPHOS (41, 63). Moreover, the cataplerotic flux, ensured by the redox shuttles, acts upstream of NNT; hence, the phenomenon is preserved independently of the NNT deficiency.

We have excluded the predominant participation of other mechanisms, hypothetically decreasing superoxide formation on GSIS (Supplementary Part II; Supplementary Fig. S2). A third one could theoretically be a switch from fatty acid to glucose metabolism (24), since fatty acid β-oxidation produces additional ROS by electron transfer flavoprotein:quinone oxidoreductase. However, since the decreasing ROS were also observed with etomoxir, an inhibitor of β-oxidation, this mechanism should not significantly contribute. The fourth mechanism would be based on the well-known elevation of cytosolic Ca^2+^ on GSIS that could be relayed by the increased Ca^2+^ uniport and compensating Ca^2+^/2Na^+^antiport ensuring the Ca^2+^ efflux (Supplementary Fig. S2Bc). Since we observed the decrease in *J*_m_ rates with blocked Ca^2+^ uniporter, either the three redox shuttles are still operating or variations in mitochondrial Ca^2+^ fluxes on GSIS probably do also contribute to these declines. We may still anticipate the participation of changed Ca^2+^ fluxes on GSIS.

The fifth possible mechanism might originate from the functional malate/aspartate shuttle decreasing respiration at low glucose *versus* its slow-down at high glucose. However, without external glutamine or on silencing of the aspartate/glutamate carriers, the decrease in the mitochondrial matrix superoxide release and accumulated superoxide/H_2_O_2_ was maintained after glucose intake. Moreover, the malate/aspartate shuttle (Fig. 1B) cannot proceed simultaneously with the three redox shuttles of Figure 1A. Nevertheless, its elimination under the low-glucose conditions (insulin nonstimulating) led to higher oxidative conditions, indicating a higher superoxide formation at retarded metabolism.

The finding of decreased mitochondrial superoxide formation on GSIS is itself a remarkable fact. This is a great phylogenetic discovery that mitochondria, representing the ATP branch of the glucose sensor in pancreatic β cells, do not acutely contribute to oxidative stress while the sensor is functioning. Perhaps this allows β cells to afford a much lower cytosolic antioxidant buffer capacity than the other cell types (30). However, since matrix NADPH_F_ decreases on GSIS, which might decrease matrix GSH, these repetitive transient decreases might contribute to oxidative stress. One can consider β cells as perfect redox machines, since they are rich in disulfide reductase-based antioxidant defenses (27). This, together with a lowered antioxidant buffer capacity allows fine redox signaling (50), on insulin secretion stimulated with glucose and branched-chain ketoacids (48). We should pay attention to these intermittent declines in the matrix antioxidant capacity, similar to the accumulated oxidative stress amplified by lipotoxicity, and glucotoxicity, which are major factors in the development of type 2 diabetes [reviewed in Aon *et al.* (1), Ivarsson *et al.* (27), Ježek *et al.* (28), Lombard and Zwaans (41), and Plecitá-Hlavatá *et al.* (49)].

In the vicinity of the Complex I flavin I_F_ site, superoxide formation increases at higher NADH/NAD^+^; otherwise, superoxide formation decreases (40, 65). Interestingly, while probing superoxide formation sites with suppressors of electron leak at the specific Complex I or Complex III ubiquinone-binding sites (9), the S3QEL suppressor diminished superoxide release into the mitochondrial matrix more intensively with increased glucose (down to ∼40% at 25 m*M* glucose). Thus with maximum glucose, substantial superoxide formation (up to 60% of the matrix-released superoxide) takes place within the outer site III_Qo_. At this site, superoxide is also formed in the presence of antimycin A. This reflects the existence of fast electron flow at elevated respiration, which is, however, not matched by the sufficient capacity of cytochrome *c* shuttling. As a result, the electron flow is retarded at the III_Qo_ site and allows superoxide formation. Since there is an increasing fraction of superoxide ascribed to the III_Qo_ site with the increasing glucose, this means that the remaining fraction, where the Complex I I_F_ site contributes, is higher at lower glucose and therefore at higher NADH_F_/NAD^+^_F_. In contrast, the Complex I I_F_ site contribution is lower at high glucose. Consequently, only the fraction of matrix superoxide release formed at the I_F_ site decreases with the acute increase in glucose in β cells. The fraction given by the III_Qo_ site then logically increases.

## Materials and Methods

### Materials

Reagents, including CitC inhibitors BTC and 4-Chloro-3-[[(3-nitrophenyl) amino] sulfonyl]-benzoic acid (CTH), were from Sigma Aldrich (St. Louis, MO), unless stated otherwise. S1QEL and S3QEL were from Life Chemicals (shipped from Spoluka Chemical Company, Kiev, Ukraine).

### Cell and PI cultures

Rat insulinoma INS-1E cells (kindly provided by Prof. Maechler, University of Geneva or purchased from AddexBio, San Diego, CA; cat. No. C0018009) were cultured in RPMI 1640 medium supplemented with 11 m*M* glucose, 5% (v/v) fetal calf serum, 10 m*M* HEPES, 1 m*M* pyruvate, 50 μ*M* mercaptoethanol, 50 IU/mL penicillin, and 50 μg/mL streptomycin (55). The 1-, 2-, or 15-h incubations were performed in RPMI 1640 with 3 m*M* glucose to lower glucose and reduced beneficial autocrine effects (51). CitC, ME1, pyruvate carboxylase, SLC25A12/AGC1/aralar, SLC25A13/AGC2, NNT, and IDH2 silencing were performed by transfections, facilitated by RNAiMax (Thermo Fisher Scientific, Waltham, MA), with properly predesigned siRNAs (Sigma), that is, for CitC (SASI_Rn01_00120045 and SASI_Rn01_00120046); ME1 (NM_012600, SASI_Rn02_00259753 and NM_012600, SASI_Rn02_00259754); pyruvate carboxylase (NM_012744, SASI_Rn01_00101243); SLC25A12/AGC1/aralar (XM_342445, SASI_Rn02_00394279 and SASI_Rn02_00394280); and SLC25A13/AGC2 (XM_001054092, SASI_Rn02_00274696 and SASI_Rn02_00274697), likewise SASI_Rn01_00066518 and SASI_Rn01_00066519 for NNT and SASI_Rn01_00093144 and SASI_Rn01_00093145 for IDH2. PIs were isolated from C57BL/6J mice (or when indicated from C57BL/6N mice) and used immediately for measurements. Nevertheless, they could be maintained for up to 1 week in the transient culture as previously described (17, 19, 60).

### Confocal microscopy assay of surplus superoxide matrix release

A Leica TCS SP2 AOBS, or alternatively Leica TCS SP8, was employed for the MitoSOX Red (Thermo Fisher) monitoring of *in situ* superoxide surplus release to the mitochondrial matrix by using 514 nm excitation and 610–679 nm emission. Rates, that is, integral fluorescence intensity increases (*J*_m_) with time, were derived in the region of interest, which was the mitochondrial network. Note that this method is feasible for the semi-quantification of mitochondrial superoxide release rates even at low or collapsed Δ*Ψ*_m_, since MitoSOX Red permanently intercalates into mitochondrial DNA (mtDNA) and cannot leak out (see also the Supplementary Data) (18).

However, even when surveying the rates, this method is frequently criticized and it is believed that it is unable to account for matrix superoxide changes (36, 67, 68). Indeed, if MitoSOX Red molecules were freely membrane permeable and not bound to mtDNA, fluorescence signal changes would potentially originate from the changes of either inner mitochondrial membrane (IMM) potential Δ*Ψ*_m_ or plasma membrane potential Δ*Ψ*_p_. However, as shown in the Supplementary Data, three aspects support the independence of the two potentials Δ*Ψ*_m_ and Δ*Ψ*_p_ under certain conditions that are suitable for feasible MitoSOX Red-based confocal microscopy assays. This is valid even for pancreatic β cells, where both potentials definitely change on the addition of glucose to cells preincubated in a medium containing lower glucose concentration. On glucose stimulation of β cells, the IMM potential Δ*Ψ*_m_ slightly increases, whereas the blockage of K_ATP_ stops the hyperpolarizing current at the plasma membrane and the subsequent burst of action potential depolarizes Δ*Ψ*_p_ to at least zero (2, 52, 55).

After their addition to cells, free membrane-permeant hydrophobic MitoSOX Red cationic molecules would redistribute between medium and cell cytosol according to the plasma membrane potential Δ*Ψ*_p_. In addition, MitoSOX Red cations would redistribute between the mitochondrial matrix and cytosol according to Δ*Ψ*_m_. Since Δ*Ψ*_m_ usually amounts to ∼180 mV and Δ*Ψ_p_ >* 60 mV, one might expect four orders of magnitude MitoSOX Red accumulation in the mitochondrial matrix space relative to the medium. After the addition of glucose, such an accumulation and hence fluorescence signal may decrease at least 10-fold due to plasma membrane depolarization, if the other aspects are not considered.

However, due to the first aspect being considered, that is, that in β cells Δ*Ψ*_m_ increases after the glucose addition (60), it would be reasonable to expect a Δ*Ψ*_m_ increase of no more than 10 mV. Hence, MitoSOX Red cation accumulation will be additionally ∼1.5 times higher in the mitochondrial matrix after glucose addition. This must lead to a much smaller decrease in the fluorescence signal, even for the freely penetrating cation.

The second aspect stems from the absence of super-resolution for this confocal microscopy assay. Conventional confocal microscopy does not afford a better resolution than 200 nm, neither 20 nm, which would be required to resolve cristae, because mitochondrial ROI (sections of mitochondrial network tubules) in conventional confocal images represent a mixture of signals originating from the intracristal space (freely accessible for small compounds from the cytosol) and the mitochondrial matrix space (due to the “zebra” of cristae resulting from mitochondrial network tubule optical sections) (50, 51). For cells with rich cristae, such as pancreatic β cells, the mitochondrial matrix signal readout would only account for around half of the fluorescence signal, whereas the remaining half would comprise, in fact, the cytosolic concentration of the MitoSOX Red cation. This contributes to the fluorescence background. Consequently, when the rates are not taken into the account and only the fluorescence intensity is considered, quantification is obscured.

The third aspect lies in the ability of MitoSOX Red to intercalate into mtDNA (18). The intercalated pool of MitoSOX Red typically dominates the confocal microscopy signal (18). Consequently, on certain values of intercalation fraction (see the calculation in the Supplementary Data, Supplementary Table S1), the integral fluorescence intensity within the mitochondrial ROI is almost insensitive to Δ*Ψ*_m_ (28, 29). Despite these theoretical considerations, the experimental sensitivity of MitoSOX Red fluorescence toward Δ*Ψ*_m_ changes may be even lower than predicted.

The mtDNA-intercalated MitoSOX Red pool is also completely insulated from accessing the cytosol or mitochondrial membranes, since it does not respond to very high doses of membrane-permeant hydrophobic pro-oxidants such as *tert*-butyl hydroperoxide (28). The disadvantage of this approach lies in a possible toxicity that could prevent correct long-term cell responses during their further culturing. Nevertheless, 20 min time-lapsed confocal microscopy recordings were found to not affect cells. However, repeated sampling with new coverslips and cell samples is recommended for long time intervals. In this way, snapshots of superoxide release rates are obtained, which provide insights into important physiological phenomena such as the redox initiation of the hypoxia-inducible factor signaling, that is, the peak in *J*_m_ rates occurring after 5 h of hypoxic incubation (Plecitá *et al.*, unpublished observations).

### Confocal microscopy assay of H_2_O_2_ release into the mitochondrial matrix within intact cells

The HyPer family of fluorescence probes has been developed for the selective detection of H_2_O_2_ (3, 4, 38, 54). We have employed vectors encoding mito-HyPer and mito-SypHer (kindly provided by Dr. Enyedi, Semmelweis University, Hungary). INS-1E cells were transfected with either one of these vectors, with the help of Lipofectamine 2000 (Thermo Fisher Scientific) for 24 h before measurements. A Leica TCS SP8 confocal microscope was employed for the intermittent recording of integral fluorescence intensity (*F*) in the mitochondrial network ROI with excitation at 405 and 488 nm, respectively.

To completely eliminate any potential pH dependency of mito-HyPer, the entire signal (in fact the fluorescence intensity per unit or chosen constant area of mitochondrial ROI) of the H_2_O_2_-insensitive but pH-sensitive probe mito-SypHer was subtracted from the mito-HyPer fluorescence signal for both excitations at 488 and 405 nm:


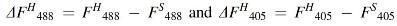


The resulting differential fluorescence data (Δ*F*) were used to calculate the ratios *R*(*H_2_O_2_*) of corrected mito-HyPer emission: *R*(*H_2_O_2_*) = Δ*F ^H^*_485_**/**Δ*F ^H^*_405_. The relative *J*_m_^H2O2^ rates were taken as the slopes of the time course for *R*(*H_2_O_2_*):


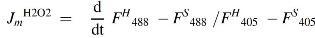


The typical resulting traces for *R*(*H_2_O_2_*) *versus* time are shown in Figure 3I–K. *R*(*H_2_O_2_*) increased significantly on the addition of a 200 μ*M* H_2_O_2_ aliquot, confirming the correct mito-HyPer response (15, 54). A *J*_m_^H2O2^ elevation was also detected with the addition of rotenone (Fig. 3K, L) or antimycin A (see Supplementary Data) and decreased after the subsequent addition of the mitochondrial matrix antioxidants SkQ1 or S1QEL. The slopes of the derived dependencies *J*_m_^H2O2^ were taken as upper limits of the observed rates and were converted to approximate values in nmols·s^−1^ by the known extent of changes after the addition of H_2_O_2_ aliquots. Such calibration is approximate due to the fact that not all added external H_2_O_2_ penetrates into the mitochondrial matrix and influences the probes localized there.

### MitoB LC-MS assay of mitochondrial ROS

The mitochondrial matrix-targeted H_2_O_2_-specific probe MitoB was used to quantify accumulated ROS in the mitochondrial matrix over time, using an adopted method (14). The boron-containing MitoB is oxidized in the mitochondrial matrix to MitoP, and both species are quantified by LC-MS. MitoP/MitoB ratios are then taken as proportional to H_2_O_2_ (ROS) accumulated within the timeframe of the experiment (2 h in our case).

The INS-1E cells were grown under the standard conditions described earlier and preincubated in medium containing 3 or 11 m*M* glucose, respectively, for 2 h at 37°C. Next, the medium was replaced with a fresh one but supplemented with 5 μ*M* MitoB probe (Sigma Aldrich). When indicated, glucose was increased to 25 m*M*. Cells were then incubated for 2 h at 37°C. In separate runs, 20 μ*M* mito-paraquat (Abcam) was included, which generates H_2_O_2_ within the mitochondrial matrix. Thus, we obtained a positive control.

After the treatment, 500 μL aliquots were removed from the reaction, snap-frozen on dry ice, and stored at −80°C before further processing. For MitoB and MitoP quantification, samples were thawed and 200 μL aliquots were transferred to new tubes. All samples were spiked with 500 n*M* internal standards of d_15_-MitoB and d_15_-MitoP (Cayman Chemicals) and vortexed for 30 s. Fifty microliters of 100% acetonitrile/0.1% formic acid (vol/vol) was added; the samples were vortexed again for 30 s, and they were centrifuged for 10 min at 16,000 *g* at room temperature. Subsequently, 100 μL sample aliquots were used for LC-MS analysis.

Mass spectra were obtained by using a Shimadzu Prominence system consisting of a DGU-20A3 mobile phase degasser, two LC-20AD solvent delivery units, an SIL-20AC cooling autosampler, a CTO-10AS column oven, SPD-M20A diode array, and LCMS-2020 mass detectors with a single quadrupole equipped with an electrospray ion source (Shimadzu, Kyoto, Japan). Binary gradient elution was used as follows: mobile phase A = water, 0.1% formic acid; mobile phase B = 100% acetonitrile; linear gradient: 0 min 30% B, 6 min 60% B; and 7 min 30% B, 10 min stop. The flow rate was 0.4 mL·min^−1^ at 25°C, and the injection volume was 10 μL.

The MS parameters were as follows: Positive mode was used, whereas the ESI interface voltage was 4.5 kV; detector voltage was 1.15 kV, the nebulizer gas flow was 1.5 mL·min^−1^, drying gas flow was 15 mL·min^−1^, heat block temperature was 200°C, DL temperature was 250°C, and the SIM mode was 397 for Mito B [M+H]^+^, 369 for Mito P [M+H]^+^, 412 for Mito B deut. [M+H]^+^, and 384 for Mito P deut. [M+H]^+^. The software LabSolutions version 5.75 SP2 was used for data quantification. The ratio of MitoP/MitoB was estimated from the respective areas under the curve obtained by the MS analysis.

### Enzymatic NADPH assay

Total cell NADPH was quantified by using a kit (BioVision, Milpitas, CA).

### Confocal microscopy assay of cytosolic and matrix NADPH

The iNAP family of NADPH-selective fluorescence probes discriminating between NADPH and NADH was developed by Dr. Yi Yang (East China University of Science and Technology, Shanghai, China) (64), who kindly provided us with iNAP1 for cytosolic and iNAP3 for matrix monitoring and the nonresponding control probe iNAPc. A Leica TCS SP8 confocal microscope was used for the intermittent recording of integral fluorescence (*F*) within ROI of each individual cell (up to 15 cells in total) with excitation at 405 and 488 nm. The *F*_405_*/F*_488_ ratios derived from each individual cell were then averaged. Since these ratios are directly proportional to cytosolic or mitochondrial matrix NADPH concentration [NADPH]_c_ or [NADPH]_m_, respectively, the average elevation after the addition of 25 m*M* glucose Δ[NADPH]_c_ or Δ[NADPH]_m_, respectively, was set as 100% and elevations in Δ[NADPH]_c_ or Δ[NADPH]_m_ relative with the tested agents were expressed normalized to this average (100%) change in their absence.

### Double-channel FLIM assay for separation of NADPH and NADH and estimation of NAD^+^

A Coherent Chameleon Ultra I mode-locked Ti:sapphire laser with 140 fs pulse width was used in conjunction with a Leica TSC SP8 confocal microscope and an attachment for 2chFLIM (Becker & Hickl, Berlin, Germany). The laser was tunable from 690 to 1040 nm with 2.9 W of average power at the peak of the tuning range, which provides ∼500 mW at 980 nm. To assay NAD(P)H by autofluorescence *in situ*, two-photon confocal excitation was set up as follows: Both NAD(P)H and FAD were excited at 700 nm whereas NAD(P)H was detected at 467–499 nm emission and time-resolved fluorescence decay yields were obtained by iterative re-convolution using SPCImage (Becker & Hickl) as two lifetimes for free and bound molecules τ_F_ = 0.4–0.5 ns and τ_B_ ∼2.6 ns, respectively (6), and their weight coefficients α_F_ and α_B_ (normalized amplitudes for which α_F_ = 1 − α_B_), respectively. Average lifetimes integrated over ROI encompassed either the mitochondrial network or extra-mitochondrial compartments or nuclei in each separate coverslip. Only a few cells were taken for calculation. The FAD was detected at 500–550 nm emission, yielding typically shorter lifetimes for bound molecules τ^FAD^_B_ and long lifetimes for free τ^FAD^_F_ (6, 26). Fluorescence lifetime τ^FAD^_F_ has previously been found to decrease in the presence of NAD^+^ because of fluorescence quenching (26).

The general NAD(P)H fluorescence decay *I*(*t*) can be expressed as:


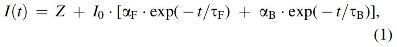


where the weight coefficients α_F_, α_B_ reflect a mixture of NADH and NADPH, *t* is time in ns, and *Z* and *I*_0_ are constants. We assumed that roughly the same lifetimes exist for NADH and NADPH, and that lifetimes are longer for bound compounds, despite the usual NADPH lifetime increases to higher values (6). This assumption represents the first simplification made in our evaluations, which yields reasonable estimates under the specific conditions when NADPH is nearly constant. Then, eq. {1} can be rewritten as:


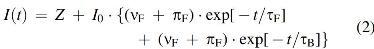


The fraction coefficients ν_F_ and ν_B_ refer to free and bound NADH species, respectively, whereas π_F_ and π_B_ refer to free and bound NADPH molecules, respectively. Assuming that the ratio ρ = π_B_/ν_B_ can be set proportionally to the concentration ratio of respective bound species (averaged from multiple binding sites on various proteins), ρ can be expressed according to the protocol devised by Duchen's group (6) as (time in ns):


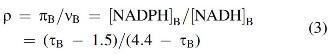


Due to the normalization of these coefficients and since by definition α_B_ = ν_B_ + π_B_, it is also valid to imply:


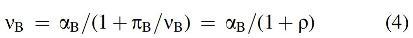


On the basis of the Scatchard equation, assumed for multiple protein binding sites, one can estimate the average free NADH concentration as:


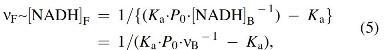


where *K*_a_ is the average association constant, and *P*_0_ is the protein concentration. By substituting eqs. {3} and {4} into eq. {5}, we obtain the following expression:


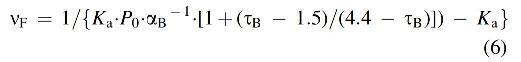


The second major simplification in our estimates takes into account the prevailing Complex I contribution so that the parameters *K*_a_ and *P*_0_ correspond to the values obtained in the kinetic model of Markevich and Hoek for Complex I, 0.02 μ*M*^−1^ and 4541 μ*M*, respectively (45). For this specific case, it is valid to presume that:


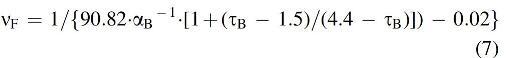


In addition, the two-photon excitation fluorescence intensity *F*(t) can be expressed (26) as:


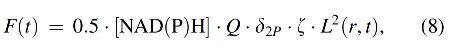


where *Q* is the quantum yield, *δ*_2P_ is the two-photon excitation fluorophore cros-section, *ζ* is the instrumental fluorescence collection efficiency, and *L*^2^(*r*,*t*) is the average spatiotemporal profile of the excitation laser pulses (6). Consequently, [NAD(P)H] can be estimated from eq. {8} by substituting *δ*_2P_ = 0.05 and *Q* = 0.02 (26) to yield:


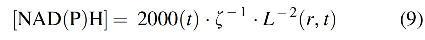


Assuming a very short time interval (shorter than the shortest lifetime) and supposing that the NADH signal can be separated from that of NADPH, we derive free NADH concentration as:


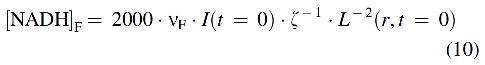


Consequently, one can estimate the change in free NADH concentration, [NADH]_F_, under two distinct conditions (*1*) and (*2*) to be:


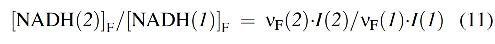


Moreover, the free NAD^+^ concentration, [NAD^+^]_F_, can be derived from the known intensity of FAD quenching by NAD^+^. The fluorescence lifetime τ^FAD^_F_ for free FAD, which is now the long-lived component (26), has been previously found to decrease in the presence of NAD^+^ due to fluorescence quenching. Assuming Stern-Volmer (*i.e.*, dynamic) quenching, it is valid to postulate that:


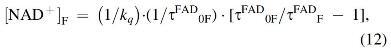


where *k*_q_ is the quenching constant, τ^FAD^_0F_ is the lifetime in the absence of a quencher, and τ^FAD^_F_ is the long-lived lifetime of FAD emission decay. For FAD, we have assumed that τ^FAD^_0F_ converges to 2.9 ns. Quenching also affects bound FAD, for which the following relationship can be proposed:


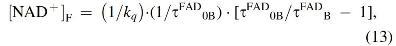


Bearing in mind that the two simplifications mentioned earlier were made, one can evaluate the substrate pressure *S* for free compounds as:


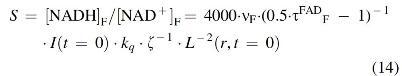


For relating substrate pressure to two distinct conditions (*1*) and (*2*), the constants involved do not need to be known and the following expression can be derived:


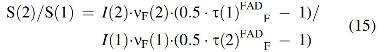


To estimate the substrate pressure for bound compounds, we use eq. {3} and the Scatchard equation for NAD^+^ and obtain:


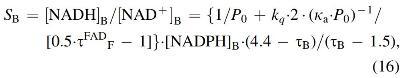


where *κ*_a_ is the association constant for NAD^+^, 0.001 μ*M*^−1^ (45). Assuming *k*_q_ = ∼0.01, the bound substrate pressure can be approximated as:


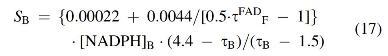


We appreciated a certain degree of precision and reproducibility of 2chFLIM. The previously established method reported the NADPH_B_/NADH_B_ ratio for bound species (6). Ultimately, 2chFLIM finally allows a complete set of parameters to be determined. Specifically, we employed the huge “background” of free FAD emission decay given by the longest-lifetime component to estimate nonfluorescent NAD^+^ due to the ability of NAD^+^ to quench FAD.

### Approximate calibration of 2chFLIM

We first confirmed the Stern-Volmer dynamic quenching relationship (eq. {12}) with 1 m*M* FAD in glycerol by additions of 50 μ*M* NAD^+^ aliquots, surveying the decreasing lifetime of FAD emission decay, approximated by a single decay component τ, using our Leica TSC SP8 confocal microscopy system with a Becker & Hickl FLIM attachment (Fig. 7G, H, gray symbols). Then, we employed INS-1E cells permeabilized by using digitonin and either used the natural cytosolic FAD content or added defined FAD amounts reaching 10 or 100 μ*M* concentrations. Again, we added NAD^+^ aliquots and now we employed 2chFLIM exactly as for the whole cell measurements, that is, including NADH channel as well, while surveying τ_2_^FAD^ decreases (Fig. 7, empty symbols). Measurements were conducted exactly as described earlier for nonpermeabilized cells. The data approximately fit to the Stern-Volmer relationship for dynamic quenching (eq. {12}), to which also fit the τ_2_^FAD^ data (lifetimes for free FAD) from Figure 8F under the assumption that NAD^+^ was around 800 μ*M*. Note also that if this NAD^+^ concentration is overestimated and may be lower in reality, then the corresponding shift of the data would still allow fitting to a slightly different Stern-Volmer relationship (eq. {12}).

### Mitochondrial membrane potential Δ*Ψ_m_* assay

Δ*Ψ*_m_ was monitored *in situ* by using tetramethylrhodamine ethyl ester (TMRE) (Thermo Fisher Scientific) in a Fluorolog 322 spectrofluorometer (Horiba Jobin Yvon, Longjumeau, France). Excitation was at 546 nm (slit width 10 nm), and emission was at 574 nm (slit width 10 nm). Cells were incubated with 10 n*M* TMRE for 15 min. Alternatively, the ratiometric probe JC-1 indicated a Δ*Ψ*_m_ decrease, which is proportional to the decrease in the ratio of JC-1 fluorescence at 593 *versus* 537 nm (29).

### High-resolution respirometry

Cellular O_2_ consumption was measured by using an Oxygraph-2k (Oroboros, Innsbruck, Austria) after air calibration and background correction (49). Endogenous respiration was recorded first and when required, glucose at the given doses or various agents were added. Nonphosphorylating respiration and nonmitochondrial respiration were determined after the addition of oligomycin and antimycin A (both 1 μ*M*), respectively. Nonmitochondrial respiration was subsequently subtracted from all measured rates. An uncoupler, carbonyl cyanide 4-(trifluoromethoxy)phenylhydrazone (FCCP), was titrated at the end of each run to assess the maximum respiratory chain capacity. The assay was conducted either in cell culture medium or in KRH buffer (135 m*M* NaCl, 3.6 m*M* KCl, 10 m*M* HEPES, 0.5 m*M* MgCl_2_, 1.5 m*M* CaCl_2_, 0.5 m*M* NaH_2_PO_4_, pH 7.4) containing 0.1% fatty-acid-free bovine serum albumin (BSA).

Islet respiratory analysis was performed on an Agilent Seahorse XF24 analyzer according to the manufacturer's manual. Fifty islets were seeded into islet capture microplates; analysis was done in KRH media containing 3 m*M* glucose and 1% FBS. The final concentration of glucose was 25 m*M*, whereas the standard testing included sequential additions of 5 μ*M* oligomycin, 1 μ*M* FCCP, and 5 μ*M* antimycin plus 5 μ*M* rotenone.

### Quantification of ATP

An ATP Bioluminescence Assay kit HSII (Roche, Basel, Switzerland) was used to quantify ATP levels. Cells were lysed by boiling in EDTA buffer (100 m*M* Tris-Cl, 4 m*M* EDTA, pH 7.75) for 2 min. Bioluminescence was determined by using a Synergy HT luminescence microplate reader (Bio-TEK/Agilent, Winooski, VT).

For the monitoring of ATP in cytosolic or mitochondrial matrix ROI, cells were transfected with cytosolic or mitochondrial ATeam plasmids (a kind gift from Prof. Hiromi Imamura, Kyoto University, Japan) (66), using Lipofectamine 2000. ATeam fluorescence was monitored with a Leica SP5 confocal microscope in lambda scan mode (XYλ). Excitation was set to 405 nm, whereas emission was collected between 453 and 608 nm (5 nm bandwidth) with a step of 3.88 nm. Integral fluorescence intensity corresponding to the excitation maximum at ∼478 nm (or its ratio to emission maximum at ∼522 nm; that is, YFP/CFP emission ratio) is inversely related to the local ATP cytosolic or matrix concentration. The former has also excluded the ATP signal from insulin granules, since emission from their *loci* is negligible.

### Quantification of insulin release

Cells were seeded at 0.3 × 10^6^ cells/well in poly-l-lysine-coated 12-well plates 2 days before the experiment. Cells were washed with the KRH^BSA^ buffer without glucose and then incubated in KRH^BSA^ with the desired glucose concentrations. After chosen the time period, insulin levels in the media were determined by using the Rat Insulin High Sensitivity ELISA kit (BioVendor, Brno, Czech Republic) (48).

### Quantification of SOD activity

An SOD assay kit (Cayman, Ann Arbor, MI) was used to estimate SOD activity. KCN additions were performed to distinguish MnSOD activity from total SOD activity.

### Quantification of cellular metabolites and ^13^C-incorporation

Quantification of pyruvate, OAA, citrate, fumarate, and malate from INS-1E cells was performed by using gas chromatography-mass spectrometry (GC-MS). Internal standard dl-malic acid-2,3,3-*d_3_* (10 μL of 300 μg/mL) was added. The cell samples were extracted with water/methanol/chloroform (1:1:2, w/w/w) and centrifuged (3000 *g*, 5°C, 10 min). The upper polar phase was transferred to glass vials and lyophilized. Analytes were derivatized with pyridine/N,O-*bis*(trimethylsilyl) acetamide/chlorotrimethylsilane (3:2:1, v/v/v) at 60°C for 70 min.

These samples (7 μL) were directly injected in split mode into the gas chromatograph coupled to a mass spectrometer (GC 6890N, MSD 5973N; Agilent Technologies, Santa Clara, CA). The 95% methyl-, 5% phenyl-polysiloxane column (15 m × 250 μm × 0.25 μm) was used for the chromatographic separation. The initial temperature of 100°C was held for 1 min, then increased by 10°C per minute to 180°C, and finally held for 1 min. Finally, the postcolumn temperature was increased to 300°C, and this temperature was held for 2 min. The total run time of analysis was 12 min with a flow rate of helium at 1 mL/min. Ions were generated by using the electron-ionization mode at 70 eV with the ion source maintained at 230°C. Ions were measured by using SIM acquisition mode. Specific ions have been selected in verified mass spectra of metabolites: pyruvate [131], OAA [217], citrate [273], fumarate [245], 2OG [347], malate [335], and dl-malic acid-2,3,3-*d_3_* [338]. Retention times of selected metabolites were confirmed with commercial standards, and detectable specific mass ions were chosen according to their mass spectra.

### Quantification of ^13^C-incorporation

The reductive carboxylation by IDH2, a counter-Krebs cycle direction reaction consuming 2OG, was semiquantified as the percentage of surplus of accumulated ^13^C-citrate and ^13^C-malate from 1-^13^C-glutamine after 6 h (59). Internal standard malate-*d*3 was added and samples were treated and measured as described earlier. The ratios between data obtained in 20 *versus* 3 m*M* glucose were calculated after subtraction of the natural ^13^C content from the rough ^13^C amounts related as the percentage of ^12^C+^13^C amount for each sample; and then the respective ratios were subsequently calculated. Thus, for malate, 2OG, and citrate (C4, C5, and C6 compounds) the derivatized fragmented ions 335, 348, and 273 (alternatively 465) contain altogether 12, 13, and 11 (alternatively 17) carbons, respectively. Hence, their natural ^13^C content is 13.2%, 14.3%, and 12.1% (alternatively 18.7%), respectively. Thus, for incorporation into malate, ^13^C-labeled malate ions 336 were traced *versus* ions 335; 349 *versus* ions 348 were traced for 2OG; as well as citrate originating ions 274 (alternatively 466) were compared with ^12^C citrate ions 273 [465].

### Statistical analysis

Analysis of variance was carried out with subsequent pairwise multiple comparisons (Tukey's or Holm-Sidak's test) by using SigmaStat 3.1 (Systat Software, San Jose, CA). Error bars in figures represent standard deviations.

## Supplementary Material

Supplemental data

## Abbreviations Used

β-OHB: β-hydroxybutyrate
2chFLIM: two-channel fluorescence lifetime imaging microscopy
2OG: 2-oxoglutarate
2OGC: 2-oxoglutarate carrier
2OGDH: 2-oxoglutarate dehydrogenase
6AN: 6-aminonicotinamide
AA: acetoacetate
ACAA1, ACAA2: acetyl-CoA acyltransferase 1, 2
ACAT1, ACAT2: acetyl-CoA acetyltransferase 1, 2
AcCoA: acetyl-CoA
ACL: ATP-citrate lyase
ANOVA: analysis of variance
AOA: aminooxyacetic acid
ASCT: alanine serine cysteine transporter
BDH: β-hydroxybutyrate dehydrogenase
BSA: bovine serum albumin
BTC: 1,2,3-benzene-tricarboxylate
Ca_L_: voltage-gated L-type Ca^2+^ channels
CitC: citrate carrier
CS: citrate synthase
CTH: 4-chloro-3-[[(3-nitrophenyl) amino] sulfonyl]-benzoic acid
CuZnSOD: copper-zinc superoxide dismutase
FA: fatty acid
FASN: fatty acid synthase
FCCP: 4-(trifluoromethoxy)phenylhydrazone
FH: fumarate hydratase
GC-MS: gas chromatography-mass spectroscopy
GDH: glutamate dehydrogenase
Gln C: glutamine carrier
GLUT: glucose transporter
GSH: reduced glutathione
GSIS: glucose-stimulated insulin secretion
HMG-CoA: hydroxymethyl-glutaryl-CoA
HMG-CoA L: hydroxymethyl-glutaryl-CoA lyase
IDH1: isocitrate dehydrogenase 1
IDH2: isocitrate dehydrogenase 2
IDH3: isocitrate dehydrogenase 3
IMM: inner mitochondrial membrane
K_ATP_: ATP-sensitive potassium
KRH: Krebs-Ringer HEPES buffer
MAC: malate aspartate carrier
MDH: malate dehydrogenase
ME1: cytosolic malic enzyme
ME3: mitochondrial malic enzyme, NADP^+^ dependent
MnSOD: manganese superoxide dismutase
mtDNA: mitochondrial DNA
NNT: nicotinamide nucleotide transhydrogenase;
NOX4: NADPH oxidase 4
LC-MS: liquid chromatography–mass spectrometry
OAA: oxaloacetate
OXPHOS: oxidative phosphorylation
PC: pyruvate carboxylase
PDH: pyruvate dehydrogenase
PI: pancreatic islet
PPP: pentose-phosphate pathway
PyrC: pyruvate carrier
ROS: reactive oxygen species
ROI: regions of interests
RR: ruthenium red
S1QEL: suppressor of complex 1 site Q electron leak
S3QEL: suppressor of complex 3 site Q electron leak
S-CoA: succinyl-CoA
SCoA:3oxoAcCoA T: succinyl-CoA:3-ketoacid-CoA transferase
SD: standard deviation
SDH: succinate dehydrogenase
siRNA: small interfering RNA
SN2: system N transporter 2
SOD: superoxide dismutase
SUCC: succinate
TMRE: tetramethylrhodamine ethyl ester
