# Supplementary material for: Mitochondrial Superoxide Production Decreases on Glucose-Stimulated Insulin Secretion in Pancreatic β Cells Due to Decreasing Mitochondrial Matrix NADH/NAD+ Ratio
Source: Antioxid Redox Signal. 2020 Sep 10;33(12):789–815. doi: 10.1089/ars.2019.7800 (PMC7482716; doi:10.1089/ars.2019.7800)
Supplement: Supplemental data [file Supp_Data.pdf]

Part I. Supplemental Results – Interpretation of Results with Partial/Complete Block of Redox Shuttles

Cytosolic malic enzyme deficiency

Cytosolic malic enzyme ME1 participates in two shuttles in the *pyruvate/malate* (*green*) and *pyruvate/citrate* redox shuttle (*magenta*) (Fig.1A; Fig.S1Aa,b). ME1 deficiency (X) alone or ME1 plus CitC deficiency (*dashed magenta line*) largely abolishes the decline of the mitochondrial superoxide release to the matrix at high glucose (Fig.4D), i.e. restores the superoxide formation; reverses the decline in the 2hr-accumulation of ROS (at least partly after 2hr-preincubation with 3 mM glucose) (Fig.4K,L); and restores parameters of 2chFLIM (Fig.8B,C,E–I). Hence, we can conclude that ME1 deficiency results in restoration of NADH formation by malate dehydrogenase, while the additional CitC deficiency (*X*) may also restore a higher NADH formation by isocitrate dehydrogenase 3 (not indicated). Resulting high substrate pressure NADH/NAD$^+$ evokes higher superoxide formation at Complex I I_F site.

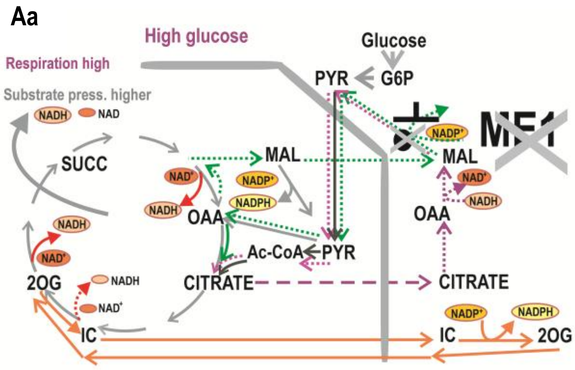

Fig. S1Aa. Metabolic fluxes upon ME1 silencing – Dotted lines represent minor or absent metabolic fluxes; solid lines existing ones. Larger size of NADH or NAD$^+$ represents higher rates of their formation. *Green*: pyruvate/malate shuttle; *magenta*: pyruvate/citrate redox shuttle; *orange*: pyruvate/isocitrate redox shuttle.

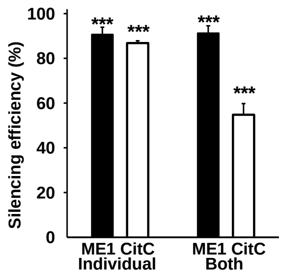

Fig.S1Ab. Silencing efficiencies for the ME1 or CitC silencing alone and for simultaneous ME1 plus CitC silencing evaluated using qRT-PCR. Differences from controls were significant, *p* < 0.001, for at least *N*=3 biological replicates (different experiments).

Ab

Pyruvate carboxylase deficiency

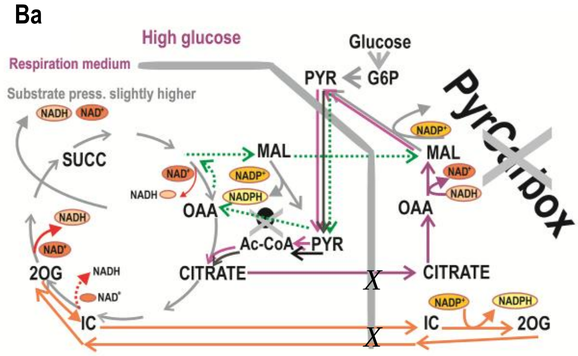

The deficiency of pyruvate carboxylase (X) abolishes the pyruvate/malate redox shuttle but leaves the other two shuttles operating (Fig.S1Ba,b). Evidently these two shuttles (*pyruvate/citrate* and *pyruvate/isocitrate* redox shuttle) are still able to cause the decline of the mitochondrial superoxide release to the matrix at high glucose, but not when CitC is simultaneously inhibited (*X*) (Fig.4C), which is blocking all three shuttles.

Fig. S1Ba. Metabolic fluxes upon silencing of pyruvate carboxylase – Dotted lines represent minor or absent metabolic fluxes. Solid lines show the operating metabolic shuttles. *Green*: pyruvate/malate shuttle; *magenta*: pyruvate/citrate redox shuttle; *orange*: pyruvate/isocitrate redox shuttle.

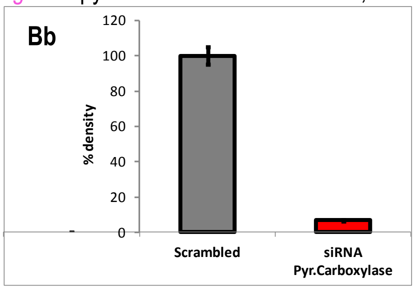

Fig.S1Bb. Silencing efficiencies for silencing pyruvate carboxylase evaluated using qRT-PCR. Differences from controls were significant, p < 0.001, for at least *N*=3 different experiments (biological replicates).

Citrate carrier inhibition

Inhibition of CitC, blocking two shuttles out of three, i.e. *pyruvate/citrate* and *pyruvate/isocitrate* redox shuttle, should also largely prevent the decline in the mitochondrial superoxide release to the matrix at high glucose, if this is caused by the redox shuttles (Fig.1A, Fig.S1Ca). Indeed, this was observed (Fig.4H), as well as the reversal of 2chFLIM parameters (Fig.8, except of panel E) and the partial reversal of the 2-hr-accumulation of ROS (Fig.4I). Hence, we can conclude that the CitC inhibition results in restoration of NADH formation by isocitrate dehydrogenase 3 as indicated Fig.S1Ca. Resulting high substrate pressure NADH/NAD^+ evokes higher superoxide formation at Complex I site I_F.

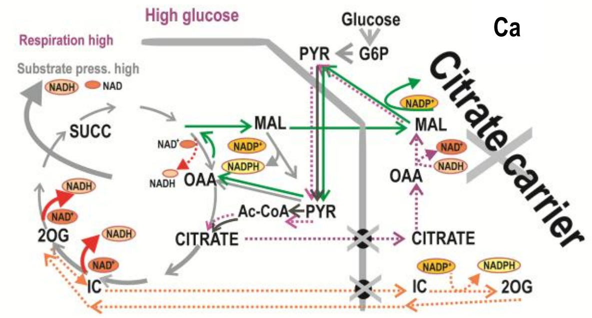

Fig. S1Ca. Metabolic fluxes upon silenced/inhibited citrate carrier – Dotted lines represent minor or absent metabolic fluxes. Solid lines show the operating metabolic shuttles. *Green*: pyruvate/malate shuttle; *magenta*: pyruvate/citrate redox shuttle; *orange*: pyruvate/isocitrate redox shuttle. X stands for silencing or inhibition.

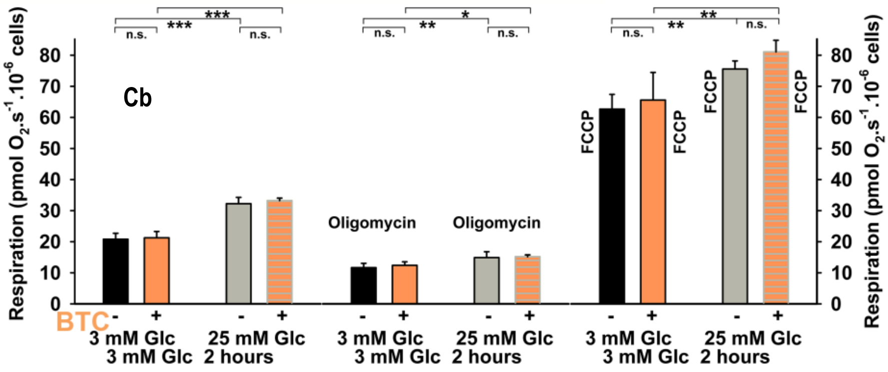

Fig. S1Cb. Respiration with BTC, an inhibitor of the citrate carrier – respiration rates of n=6 independent samples were measured for each condition, using Oroboros oxygraph 2k with or without 10 mM BTC as indicated. ANOVA: *** $p < 0.001$; ** $p < 0.01$; n.s. non-significant.

2-oxoglutarate carrier inhibition

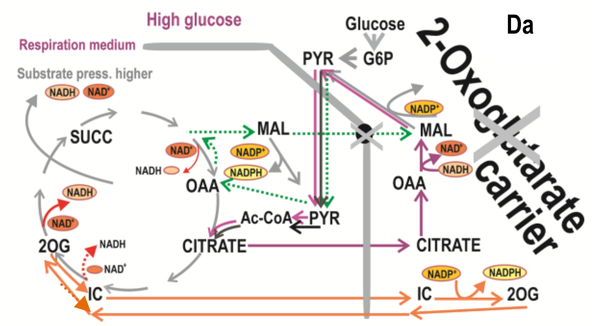

Inhibition of 2OGC principally blocks only the *pyruvate/malate* redox shuttle out of three shuttles. One might expect only a partial prevention of the decline in the mitochondrial superoxide release to the matrix at high glucose, with the *pyruvate/malate* redox shuttle participating (Fig.1A, Fig.S1D). This is indeed observed (Fig.4H). But the reversal of the 2hr-accumulation of ROS was complete (Fig.4I). 2OGC is also required to provide cycling (transport back to the matrix) of 2OG within the *pyruvate/isocitrate* cycle. Nevertheless, this can be substituted by other metabolic carriers such as dicarboxylate carrier.

Fig. S1Da. Metabolic fluxes upon silenced or inhibited 2-oxoglutarate carrier – Dotted lines represent minor/absent metabolic fluxes. Dashed line shows minor fluxes due to carrying 2OG possibly by other metabolic carriers. X stands for silencing or inhibition.

Blockage of Pentose Phosphate Pathway

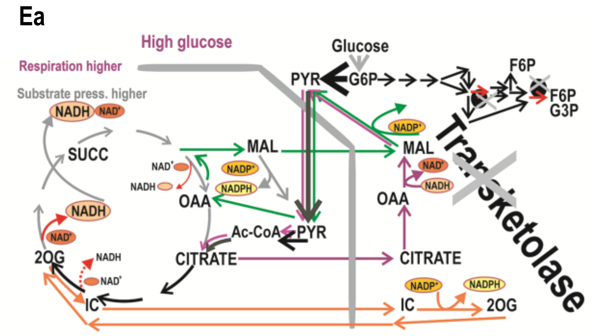

PPP blockage did not significantly affect the decline of the mitochondrial superoxide release to the matrix at high glucose (Fig.4C), neither decline in 2hr-accumulation of ROS (Fig.4D). The latter proceeded from higher oxidative state (Fig.S1Ea,b).

Fig. S1Ea. Metabolic fluxes upon inhibition of transketolase with oxythiamine, i.e. block of the pentose phosphate pathway – PPP is illustrated schematically. As a result of its block, a higher pyruvate flux is allowed to enter for pyruvate dehydrogenase and pyruvate carboxylase reaction. *Green*: pyruvate/malate shuttle; *magenta*: pyruvate/citrate redox shuttle; *orange*: pyruvate/isocitrate redox shuttle

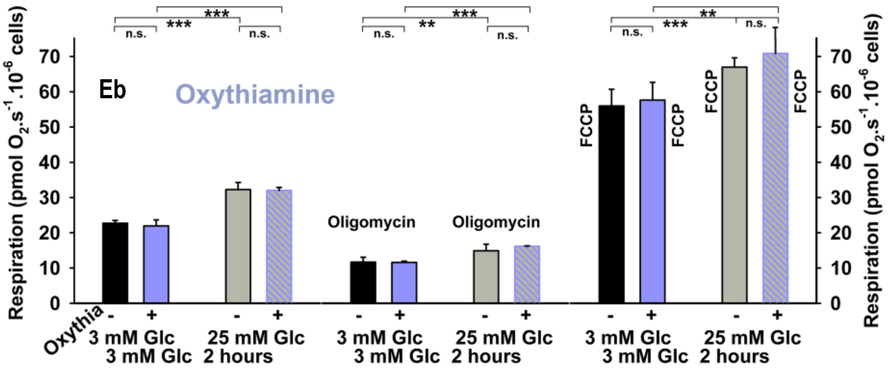

Fig. S1Eb. Respiration with oxythiamine (40 μM), an inhibitor of transketolase, and hence PPP – rates of *N*=4 cultivations were measured, employing Oroboros oxygraph 2k. ANOVA: *** p < 0.001; ** p < 0.01; n.s. non-significant.

Part II. Supplemental Results – Concurrent hypotheses and roles of NNT, IDH2 and β-hydroxybutyrate dehydrogenase reactions

Note, as the main hypothesis (**Hypothesis I**), we consider the decline of mitochondrial ROS formation upon GSIS as resulting from the operation of the three redox shuttles, pyruvate/malate shuttle; pyruvate/citrate shuttle; and pyruvate/isocitrate shuttle.

Hypothesis II considers the decline of mitochondrial ROS formation upon higher OXPHOS intensity upon GSIS, meaning higher proton backflux via the ATP-synthase c-ring which effectively decreases mitochondrial superoxide formation namely within the respiratory chain.

Hypothesis III:

Enhanced β-oxidation of endogenous fatty acids at low glucose would provide higher superoxide formation relatively to high glucose:

Gerencser has suggested that elevated glucose metabolic control leading to a maximum $\Delta\Psi_m$ pre-determines optimum secretion of insulin (23). At low glucose a higher H^+-leak (uncoupling of mitochondria) may contribute to an elevated non-phosphorylating (H^+-leak-driven) respiration relatively to such a state without an H^+-leak. We did inspect this in a thorough study of INS-1E cells and demonstrated that fatty acid β-oxidation stimulates uncoupling protein UCP2 function *via* H_2O_2-activated mitochondrial phospholipase iPLA$_2\gamma$ (27). *Hypothetically, ETF:QOR-mediated surplus of mitochondrial ROS might contribute to the observed higher oxidative status of the mitochondrial matrix relatively to the situation with high insulin-stimulating glucose, where fatty acid β-oxidation would be suppressed by glucose metabolism* (Fig.S2Aa).

However, we excluded this hypothesis, since etomoxir did non-significantly diminish phosphorylating INS-1E cell respiration at 3 mM glucose as well as maximum respiration, but maintained non-phosphorylating, i.e. leak-driven respiration (Fig.S2Ab). Thus endogenous fatty acid β-oxidation does not significantly contribute under our experimental conditions, which is evidenced also by virtually unchanged parameters *R*r and *A*r with etomoxir (Fig.2G). As a result matrix superoxide release decreases similarly with etomoxir as in its absence (Fig.4A), similarly as ROS accumulated during 2 hrs assayed with MitoB (Fig.4A). We conclude that despite contribution of mechanism suggested by the Hypothesis III could be still plausible *in vivo*, it does not significantly contribute under our experimental conditions.

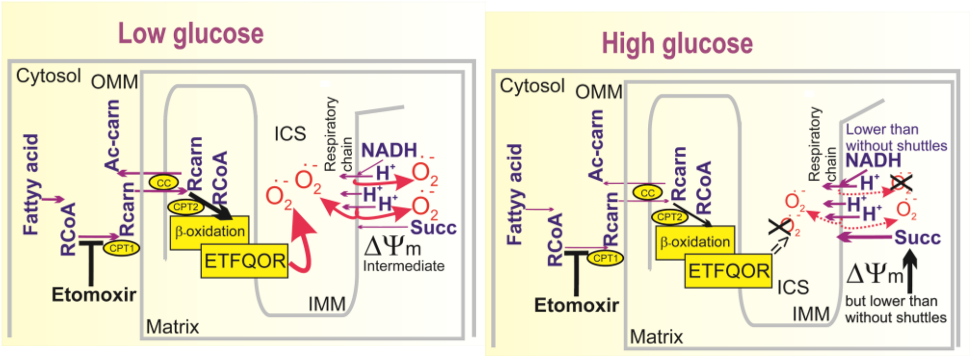

Fig. S2Aa. Hypothesis III – assumes that fatty acid- (FA-) β-oxidation is faster at low glucose than at high glucose. Since ETFQOR (electron-transfer flavoprotein : quinone oxidoreductase) of β-oxidation forms superoxide, so according to Hypothesis III, its formation would be lower at high glucose. Etomoxir, blocking carnitine palmitoyltransferase-1 (CPT1) would eliminate this. Since etomoxir still enables lower superoxide formation rate with high glucose, we must exclude Hypothesis III.

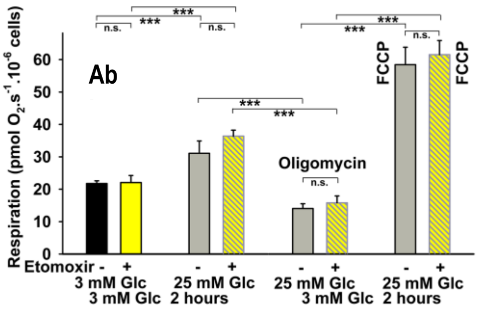

Fig. S2Ab. Respiration of INS-1E cells in the absence or presence of fatty acid β-oxidation inhibitor etomoxir – respiration rates of *n*=4–7 independent samples (*N*=2) were measured using Oroboros oxygraph 2k. Where indicated, 100 μM etomoxir was present. ANOVA: *** $p < 0.001$; n.s. non-significant.

Hypothesis IV: Elevated cytosolic Ca^{2+} upon GSIS may dysbalance mitochondrial Ca^{2+} cycling in favor to Ca^{2+} influx that would lead to decreased $\Delta\Psi_m$ and hence lower mitochondrial superoxide formation at high glucose

We attempted to exclude this hypothesis by blocking only the Ca^{2+} influx using ruthenium red or only the Ca^{2+} efflux using CGP37157 in INS-1E cell at 3 vs. 25 mM glucose (Fig.S2Ba,b,c). Despite the fact that insignificant inhibition of respiration took place with 7 μM ruthenium red (99% ± 4%) and that parameters *R*r and *A*r were slightly diminished. Also, despite the normal MitoSOX Red response (Fig.4A), 2hr-accumulation of ROS was extremely elevated with ruthenium red and higher elevation was in 25 mM glucose. We cannot therefore definitively exclude Hypothesis IV, but the obtained data did not exclude participation of the three redox shuttles investigated in this work.

Also inhibition of mitochondrial Ca^{2+} efflux using CGP37157 in INS-1E cells exhibited a non-specific effect at 25 mM glucose, most probably opening of permeability transition, suggested by the leakage of MitoSOX Red probe to the cell cytosol at 25 mM (but not at 3 mM) glucose. The concomitant collapse of mitochondrial respiration (Fig.2G) then helps to interpret the observed decline in the 2hr-accumulation of ROS, which is non-specific and does not support our main hypothesis.

Ba Ruthenium red

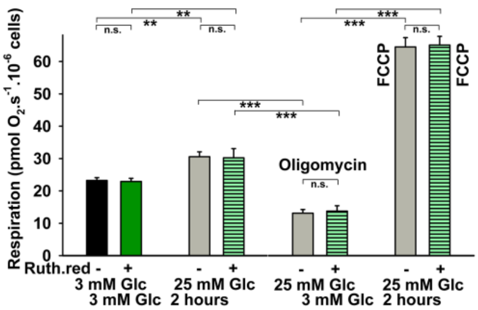

Bb CGP37157

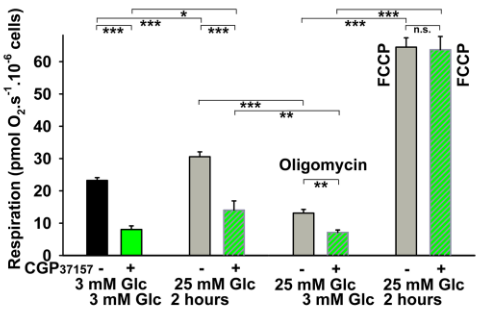

Fig. S2Ba,b. Respiration of INS-1E cells – in the absence or presence of 7 μM ruthenium red **(Bb)** and CGP37157 **(Bc)** (10 μM), i.e. blocking Ca^{2+} influx and Ca^{2+} efflux, respectively – respiration rates of *n*=4–7 independent samples for each condition (*N*=3) were measured using Oroboros oxygraph 2k. ANOVA: *** $p < 0.001$; ** $p < 0.01$; * $p < 0.1$.

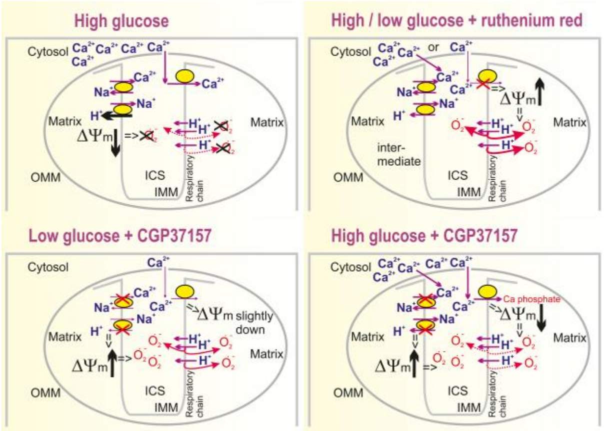

Bc

Fig. S2Bc. Cation fluxes upon GSIS and with inhibition of Ca^{2+} influx into the matrix or Ca^{2+} efflux from the matrix – *Upper right*: ruthenium red inhibits Ca^{2+} uniporter and hence Ca^{2+} influx into mitochondrial matrix. As a result, the Ca^{2+} influx no longer diminishes $\Delta\Psi_m$, hence this potential is high, which leads to the enhanced mitochondrial production of superoxide. *Lower left*: Ca^{2+}/Na^+ antiporter, which otherwise ensures the Ca^{2+} efflux, is inhibited by agonist CGP37157. The block of Ca^{2+} efflux at the simultaneous slow metabolism at low glucose allows the dysbalanced Ca^{2+} influx which slightly decreases $\Delta\Psi_m$. However, the absence of the effective uncoupling by the H^+ influx mediated by the Na^+/H^+ antiporter, which is no longer driven by the Na^+ influx mediated by the Ca^{2+}/Na^+ antiporter, leads to increase in $\Delta\Psi_m$. As a result, still higher mitochondrial superoxide formation exists. *Lower right*: The same situation at high glucose now changes since the high cytosolic Ca^{2+} occurs upon GSIS, leading to prevailing decrease in $\Delta\Psi_m$, hence mitochondrial superoxide formation is lower. Due to this massive Ca^{2+} influx, however, non-specific phenomena occur.

Hypothesis V: Participating malate/aspartate shuttle at low glucose leads to a higher substrate pressure, higher $\Delta\Psi_m$ and hence higher mitochondrial superoxide formation at low glucose; superoxide formation at high glucose is then automatically lower and does not require other redox shuttles.

This hypothesis is illustrated in Fig.1B and Fig.S2Ca. We excluded this hypothesis, showing that if any contribution of the malate/aspartate shuttle exists at low non-stimulating glucose to mitochondrial ROS formation, it is minor (Fig.4B,E; Fig.S2Cb). The blockage of the malate/aspartate by silencing of both of aspartate/glutamate carriers led only to general decrease of superoxide formation (Fig.4B). This occurred by a larger extent at 3 mM glucose and by some extent also at 25 mM glucose (Fig.4B). Thus the assumption that the malate/aspartate shuttle brings a higher substrate pressure ($NADH/NAD^+$) into the mitochondrial matrix was verified, since upon the shuttle blockage, the superoxide formation went down due to the substrate pressure decline. Nevertheless, 25 mM glucose decreased the matrix superoxide release rates (Fig.4B) and 2hr-accumulated ROS even further down (Fig.4E; Fig.S2Cb), thus excluding the malate/aspartate shuttle as a major player in this phenomenon and hence the remaining plausible hypothesis is *Hypothesis I* which must interpret these results as stemming from the function of the three redox shuttles – *pyruvate/malate*, *pyruvate/citrate*, and *pyruvate/isocitrate* redox shuttles.

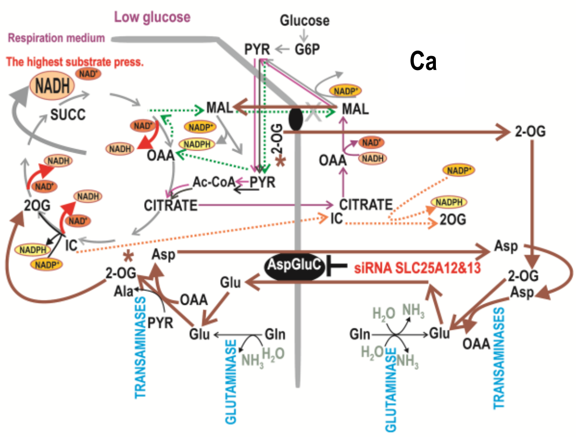

Fig. S2Ca. Malate/aspartate shuttle at low (insulin non-stimulating) glucose is depicted in *brown* color. This shuttle is bringing NADH redox equivalents to the mitochondrial matrix from the cytosol and relies on transaminases (aminotransferases) and two isoforms of aspartate/glutamate antiport carrier: SLC25A12, i.e. Aralar or AGC1; and SLC25A13, *i.e.* AGC2 (*big black ellipse "AspGluC"*). As a result, the highest established substrate pressure ($NADH/NAD^+$) induces the highest superoxide formation at the site I_F of the Complex I (see Fig.10). The fluxes through the 2OG antiporter (exchanging malate with 2OG, *black ellipse*) are thus opposite relatively to the fluxes upon the operation of pyruvate/malate shuttle (*green*), which is therefore inhibited (*dotted lines* depict non-existing or suppressed fluxes) when the malate/aspartate shuttle is operating. Upon silencing of both aspartate/ glutamate carriers or at high (insulin-stimulating) glucose the malate/aspartate shuttle should be largely suppressed by the opposite 2OG and malate fluxes upon activation of the three depicted pyruvate redox shuttle; * = connection.

Also, the decline of the mitochondrial superoxide release to the matrix at high glucose still exists upon inhibition of aminotransferases (transaminases) using AOA (Fig.S2Cc,Cd), despite this treatment inhibits respiration. Moreover, just a simple omitting of glutamine in the medium enhances this effect since 2hr-accumulation of ROS is extremely enhanced in the absence of glutamine. This again stems from the retarded respiration (Fig.S2Cc,Cd). AOA abolishes this extreme 2hr-accumulation of ROS independently of the presence of glutamine (Fig.4E).

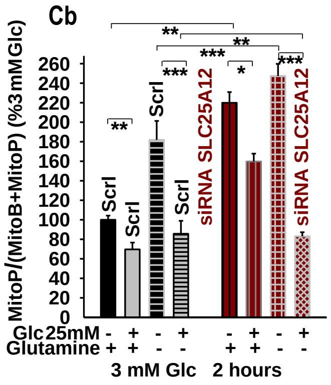

Fig.S2Cb. 2hr-ROS accumulation upon silencing aspartate/glutamate antiport carrier SLC25A12, i.e. Aralar or AGC1 as an key component of the malate/aspartate shuttle in the presence or absence of glutamine as compared to cells transfected with scrambled siRNA („Scrl"). Note, blockage of the malate/aspartate shuttle either using glutamine withdrawal or silencing SLC25A12 leads to a profound oxidative stress, that is prevented at high glucose due to operation of the three pyruvate redox shuttles. ANOVA (*N*=3): *** $p < 0.001$; ** $p < 0.01$; * $p < 0.1$. Silencing efficiency overcome 70% as evaluated using qRT-PCR, similarly as for SLC25A13 (not shown).

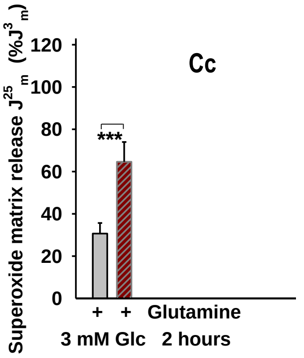

Fig. S2Cc. J_m rates: malate/aspartate shuttle blocked by inhibition of transaminase with AOA – corresponding to the observed inhibition of respiration with AOA, the observed superoxide release into the mitochondrial matrix with 25 mM glucose was then decreased by a lower extent. ANOVA (*N*=3): *** p < 0.001.

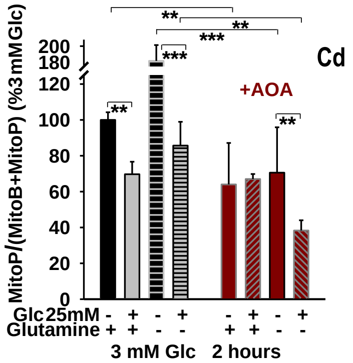

Fig. S2Cd. 2hr-ROS accumulation: malate/aspartate shuttle blocked by inhibition of transaminase with AOA – reflecting inhibition of respiration with AOA, 2hr-ROS accumulation was lower with or without glutamine. With glutamine non-specific effect and irreproducibility did not enable to observe further effect of pyruvate redox shuttles. ANOVA (*N*=3): *** p < 0.001; ** p < 0.01.

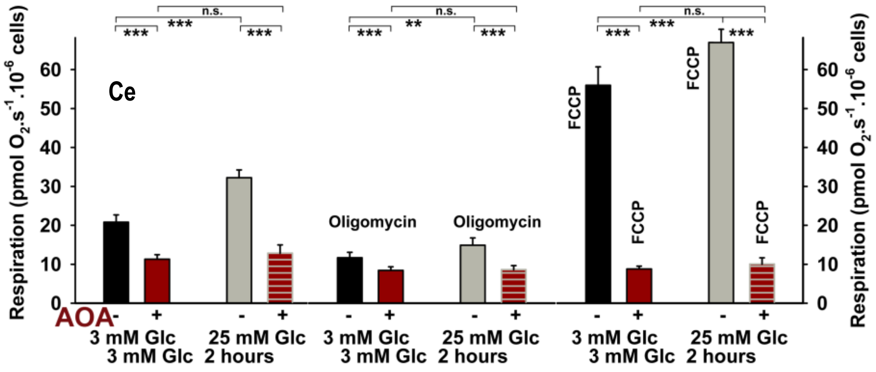

Fig. S2Ce. Inhibition of respiration with AOA – respiration rates of *n*=9 samples (*n*=4 with AOA; *N*=3) were measured using Oroboros oxygraph 2k. Where indicated, 4 mM AOA was present. ANOVA: *** p < 0.001; ** p < 0.01; n.s. non-significant.

NNT participation: Nicotinamide nucleotide transhydrogenase contributes to the matrix NADPH pool

A complex role in pancreatic β-cells is played by NNT, so that frequently controversial reports are published (58).

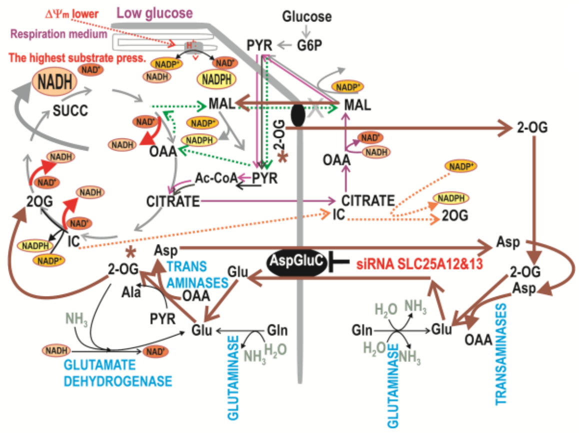

Da

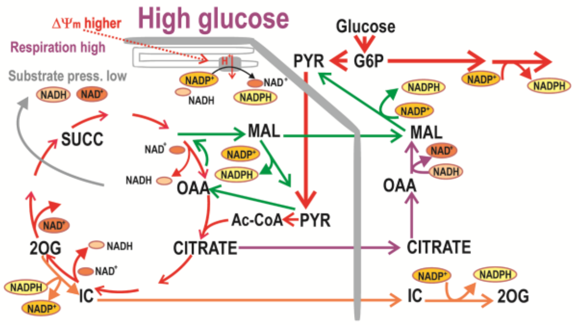

Fig. S2Da. Role of NNT – At low glucose (*left*), $\Delta\Psi_m$ is lower, hence nicotinamide nucleotide transhydrogenase, driven by Δp could theoretically reverse its mode, meaning to act as a proton pump. This might be facilitated by the increased NADPH synthesis due to the simultaneous oxidative (Krebs cycle forward) mode of IDH2. However, despite lower metabolic rate, there is still a relative NADH excess due to lower respiration (under the highest substrate pressure), hence the realistic mode of NNT has yet to be investigated. Preliminary data see Fig.2L. **At high glucose** (*right*), IDH2 produces $NADP^+$, being in the mode of reductive carboxylation as a part of the isocitrate/pyruvate shuttle. Hence, despite higher $\Delta\Psi_m$ and Δp which could drive faster NNT reaction, the NNT reaction proceeds at somewhat lower NADH levels (due to the shuttles), despite possibly higher $NADP^+$ levels. Again, the realistic mode of NNT is now the forward mode, which was supported by the data of Fig.2L.

This is, however, beyond the scope of this article. We attempted to solve the question, whether NNT acts in a forward (producing NADPH and consuming Δp) or reversed mode (consuming NADPH and ensuring a Δp surplus by proton pumping)(Fig.S2Da). Our preliminary $\Delta\Psi_m$ measurements, using JC1 fluorescence monitoring, indicated that $\Delta\Psi_m$ rose upon silencing of NNT at both 3 mM as well as at 25 mM glucose. This suggests the existence of forward NNT mode for both low and high glucose, i.e. NADH-, NADP+- plus Δp-consuming reaction, producing NAD^+ and NADPH (Fig.2L). Note that the other authors reported the reverse mode of NNT (58).

However, since NNT acts downstream of redox shuttles, NNT silencing did not profoundly altered responses of MitoSOX and MitoB probes to transitions from 3 mM to 25 mM Glucose (Fig.S2Dc; S2Dd). Also, a fraction of the mitochondrial matrix NADPH pool, to which NNT contributes, can be estimated from the comparison of NNT-silenced to scrambled INS-1E cells when $[NADPH]_m$ is monitored by confocal microscopy with the iNAP3 fluorescence probe (Fig.5E–G). Fig.S2Da shows the expected results, Fig2De the silencing efficiency. Indeed, relative drop in the iNAP3 fluorescence persists at transition from 3 mM to 25 mM glucose.

Db

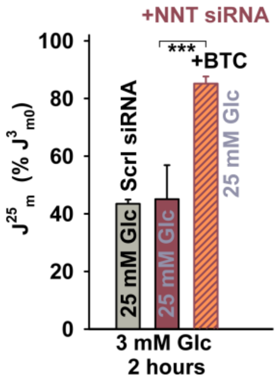

Dc

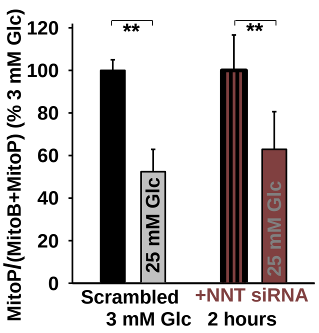

Fig. S2Dc, S2Dd NNT-silenced INS-1E cells exhibit regular slow down of superoxide/H_2O_2 release into the matrix, similar to cells transfected with scrambled ("Scrl") siRNA, as accessed using MitoSOX (**c**) and MitoB probes (**d**), respectively Where indicated, 10 mM BTC was present. ANOVA: *** p < 0.001; ** p < 0.01 (*N*=2; *n*=9) for each probe).

De

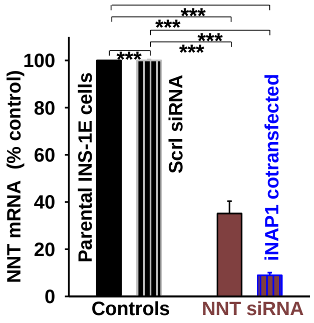

Fig. S2De Efficiency of NNT silencing in experiments of Fig.5 and Fig.S2Db–Dd. Stock INS-1E cells ("Parental"), and those transfected with scrambled siRNA ("Scrl") are compared with those transfected with sole NNT siRNA or with simultaneous iNAP1 probe overexpression (for experiment of Fig.5E using qRT-PCR. Differences from controls were significant, p < 0.001, for 3 different experiments for each condition.

IDH2 participation:

Isocitrate dehydrogenase 2 (IDH2) influencing the matrix NADPH /$NADP^+$ is part of the pyruvate/isocitrate shuttle

A complex role is also played by IDH2, which is also beyond the scope of this article, despite IDH2 being the crucial part of the pyruvate/isocitrate shuttle. As Fig.1B and Fig.S2Ea show, at low glucose (3 mM; insulin non-stimulating concentration) IDH2 is expected to act in the oxidative mode, decarboxylating isocitrate to 2OG, while consuming $NADP^+$ and contributing to the matrix NADPH pool. Upon a glucose rise to 25 mM (i.e. insulin stimulating dose) and provided being in CO_2 microenvironment, IDH2 reverses its reaction to the reductive carboxylation; now consuming NADPH and 2OG while providing $NADP^+$ and isocitrate.

This reversal is a part of the pyruvate/isocitrate redox shuttle and its existence is demonstrated in Fig.6D. IDH2 silencing (achieved efficiency see Fig2Eb) may reflect some of the features. Fig.S2Ec,d show results of MitoSOX or MitoB assay of matrix superoxide or 2hr ROS release, respectively. The superoxide release decline exists upon IDH2 silencing, but is by ~10% lower, since there are still two other redox shuttles acting. In contrast the drop in

2hr-accumulation of ROS vanished. Unchanged rise in cytosolic $NADPH_F$ upon IDH2 silencing, monitored with the iNAP1 fluorescence probe, reflected possible dominancy of ME1, (Fig.S2Ee).

Upon IDH2 silencing, the nicotinamide nucleotide equilibrium is altered even at 3 mM glucose, so the transitions from low to high glucose surveyed using the iNAP3 monitoring should be interpreted distinctly. Fig.S2Ef shows the expected results, i.e. theoretical consideration dependent on the particular NNT mode at low and high glucose. The experimental results are shown in Fig.5E,F, i.e. results of iNAP3 monitoring when comparing the IDH2-silenced to control INS-1E cells transfected with scrambled siRNA. These results confirm variant when NNT acts in the forward mode at both low and high glucose. We emphasize here that IDH2 is one of three enzymes contributing to the matrix NADPH pool (together with NNT and ME2). The observed $[NADPH_F]_m$ decrease at transition from low to high glucose upon IDH2 silencing was slightly lower and was only marginally inhibited by further addition of BTC. This stems from the non-existence of reversal to the NADPH consuming reaction at IDH2 deficiency.

Ea

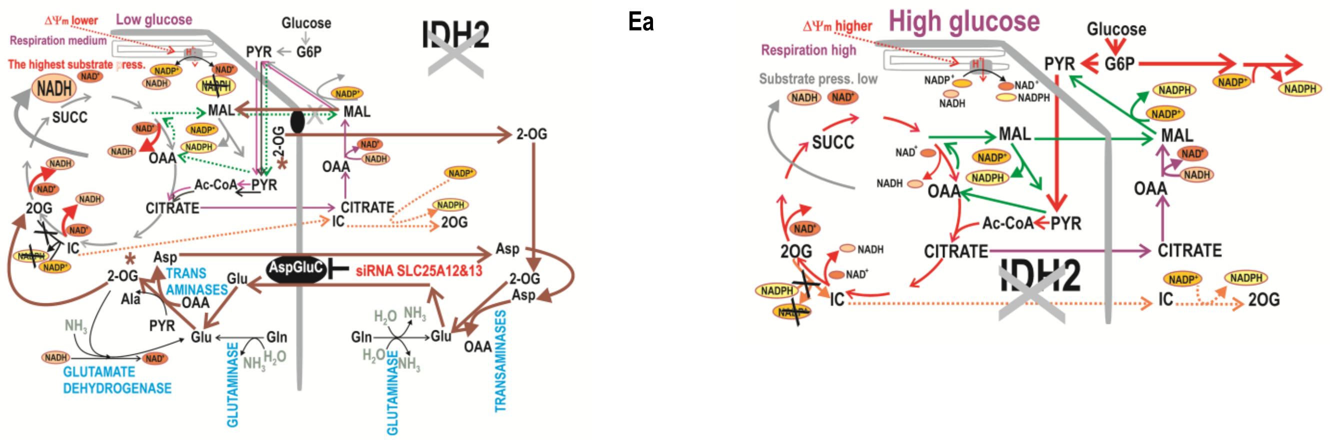

Fig. S2Ea. Effects of IDH2 silencing – At low glucose (*left*), IDH2 deficiency causes a lower overall production of NADPH in the matrix, thus not allowing reversed mode of NNT. **At high glucose** (*right*), IDH2 deficiency stops $NADP^+$ production, hence leave slightly elevated NADPH. The IDH2 deficiency simultaneously interrupts the isocitrate/pyruvate redox shuttle, however, both remaining redox shuttle should still exist.

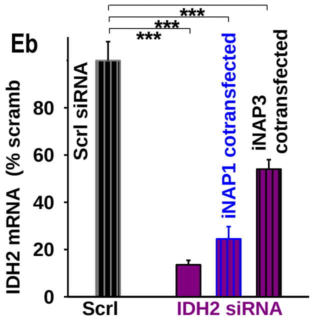

Fig. S2Eb. Efficiencies of IDH2 silencing – evaluated using qRT-PCR. Differences from controls were significant, P<0.001, for *N*=4 experiments (biological replicates).

Fig. S2Ec. Matrix superoxide release upon IDH2 silencing – measured as described for data of Fig.4. *n*=4 for scrambled siRNA transfections; *n*=9 for IDH2 siRNA transfections; *N*=2 biological replicates. Student's T-test: ** p < 0.05.

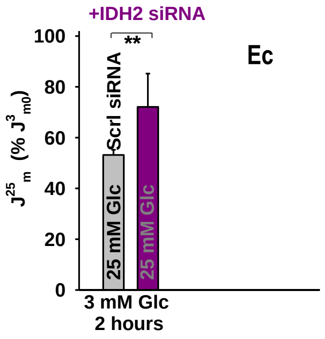

Fig. S2Ed. Superoxide/H_2O_2 2hr-accumulation In the matrix upon IDH2 silencing – in cells transfected with scrambled ("Scrl") siRNA vs. IDH2 siRNA, assessed using MitoB probe. ANOVA (*n*=3–9): *** p < 0.001.

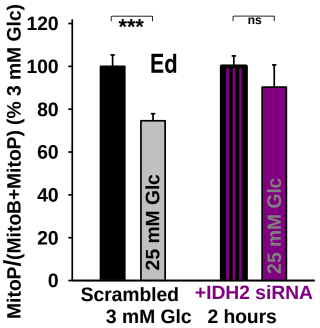

Fig. S2Ee. Cytosolic NADPH changes upon IDH2 silencing – measured using the iNAP1 fluorescence probe specific for NAPDH, as described for data of Fig.5. *N*=4 (n=60 spots of ROI) for scrambled siRNA transfections ("Scrl"); N = 7–9 (n=120–150 spots of ROI) for IDH2 siRNA transfections. ANOVA: *** p < 0.001.

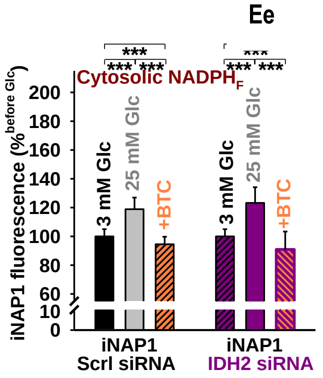

Fig. S2Ef. Theoretical considerations of outcome for IDH2 silencing at distinct NNT modes – under assumption of equal contribution to the matrix $NADPH_F$ pool (described as a single molecule). *Red numbers* ascribe the total amount of their equivalents, so to predict changes in the matrix $NADPH_F$. This model, however, does not account for the different activities of the three considered enzymes and reversals of ME3 reaction.

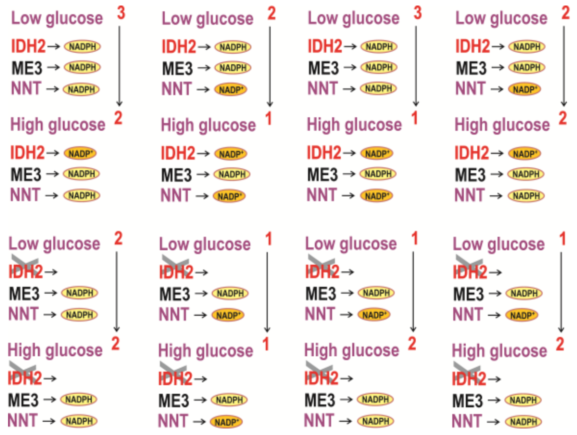

β-hydroxybutyrate dehydrogenase participation:

Several studies concerned with β-hydroxybutyrate dehydrogenase (β-OHBDH) in pancreatic β-cells (42,46), however, results of β-hydroxybutyrate concentrations are scarcely reported in numerous recent metabolomics studies (e.g. 12,51,62). Traditionally, the ratio of β-hydroxybutyrate to acetoacetate was considered to reflect relations between NAD^+ and NADH in the mitochondrial matrix (46). In light of the recent metabolomics, it is now clear that the ratio of the matrix NAD^+/NADH (actually reciprocal substrate pressure *S*) is really quite high. One may consider even values >100. In HeLa cells, absolute concentrations of 800 μM NAD^+ and 5 μM NADH were reported, accounting for the NADH/NAD^+ ratio of 0.00625 and the NAD^+/NADH ratio of 160 (12). The ratio of β-hydroxybutyrate to acetoacetate (which was supposed to be proportional to the NAD^+/NADH ratio) can unlikely be in this range. Moreover, the NADH-driven β-OHBDH reaction converting acetoacetate to β-hydroxybutyrate and NAD^+ is not the only one involving acetoacetate in pancreatic β-cells (42,46) (Fig.S2Fa). Acetoacetate was found to be transported to the cytosol of β-cells, where it reportedly facilitates insulin secretion via forming various acyl-CoA derivatives (42). Nevertheless, since β-OHBDH cannot be made in the cytosol, except in humans (see https://www.brenda-enzymes.org/enzyme.php?ecno=1.1.1.30), β-hydroxybutyrate can be still regarded as proportionally reflecting matrix NAD^+. At least, an increment in β-hydroxybutyrate upon GSIS must contribute to the increment of the NAD^+ pool in the matrix.

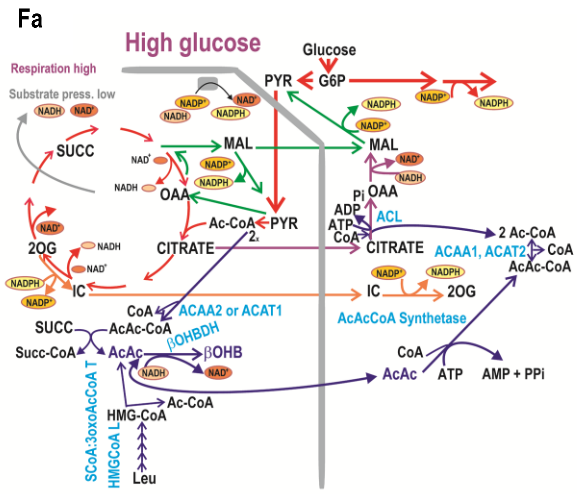

Fig. S2Fa. Theoretical considerations for β-hydroxybutyrate and acetoacetate metabolism in pancreatic β-cells – cf. Ref (42). At high glucose, sufficient succinate load enables its interconverion by succinyl-CoA:3-ketoacid-CoA transferase (SCoA:3oxoAcCoA T) with acetoacetyl-CoA (AcAcCoA) into succinyl-CoA (Succ-CoA) and acetoacetate (AcAc). This can be formed also by a Leucine metabolism, within a series of reaction of β-like oxidation leading to hydroxymethyl-glutaryl-CoA (HMG-CoA), which is split by HMG-CoA lyase (HMG-CoA L). From the matrix acetoacetate pool, acetoacetate can be easily transported to the cytosol or may be converted by the β-OHBDH to β-hydroxybutyrate at the expense of NADH, thus forming NAD^+. Note that distinct enzyme isoforms convert two molecules of Acetyl-CoA (Ac-CoA) into CoA and AcAcCoA in the mitochondrial matrix (Acetyl-CoA Acetyl- transferase, ACAT1; and Acetyl-CoA Acyl- transferase, ACAA2) and cytosol (Acetyl-CoA Acetyl- transferase, ACAT2; and Acetyl-CoA Acyl-transferase, ACAA1).

Part III. Supplemental Results – Validation of MitoSOX Red confocal microscopy semi-quantification of mitochondrial matrix superoxide release

To ensure that the MitoSOX Red based confocal microscopy semi-quantification of mitochondrial matrix superoxide release is also nearly independent of potential for insulinoma INS-1E cells, we provide the following experiments illustrating practical performance of MitoSOX Red time-lapsed fluorescence monitoring.

First, matrix superoxide release rates J_m were evaluated in the presence of ionophores that drastically change $\Delta\Psi_m$ and $\Delta\Psi_p$. We came to the conclusion that J_m rates are independent of potential. Thus the entire $\Delta\Psi_m$ and $\Delta\Psi_p$ was dissipated upon the addition of the potassium uniporter valinomycin and compared with the results of the higher $\Delta\Psi_m$ established by nigericin. Note, nigericin collapses the ΔpH component of Δp, but equally increases $\Delta\Psi_m$. Such a well-known behavior of these ionophores can be monitored by the ratiometric fluorescence probe JC-1, indicating a decrease in the ratio of JC-1 emission at 593 nm vs. 537 nm with progressively decreasing $\Delta\Psi_m$ set by increasing valinomycin doses (Fig.S3A). In contrast, increasing the nigericin concentration did not give such a response. Clearly, $\Delta\Psi_m$ was decreased by valinomycin.

The parallel estimations of J_m rates for INS-1E cells cultured in medium with 11 mM glucose and ROI set to the mitochondrial network yielded similar time courses of integral MitoSOX Red fluorescence *vs*. time for the controls, as well as for samples with collapsed $\Delta\Psi_m$ with valinomycin, and with nigericin (Fig.S3B). Irrespective of the presence of valinomycin or nigericin presence, these traces were also virtually identical for INS-1E cells preincubated with 3 mM glucose (Fig.S3C) or those enriched with 25 mM glucose (Fig.S3D). As a result we can employ our experimental semi-quantification under conditions when $\Delta\Psi_m$ varies in INS-1E cells, such as when it is elevated upon glucose addition. In conclusion, despite the lack of calibration to absolute amounts of superoxide, we can compare two situations that have distinct potential values.

Second, we have evaluated the independence of the plasma membrane potential $\Delta\Psi_p$ in our method. We thus validated the special case of INS-1E cells as a pancreatic β-cell model, when glucose is added to the cells pre-incubated with lower glucose and when $\Delta\Psi_p$ is depolarized nearly to almost zero values (1, 47, 51). Can integral MitoSOX Red fluorescence within the mitochondrial network ROI be influenced? We found that the increase in MitoSOX Red integral fluorescence with time (J_m) was equal in the absence and presence of glibenclamide at 3 mM glucose (Fig.S3E). Note that glibenclamide blocks the ATP-sensitive K^+ channels (K_{ATP}) (1, 51), resulting in $\Delta\Psi_p$ depolarization. This independence shows that the back-diffusion of the MitoSOX Red cation from the mitochondrial matrix into the intracristal space and further to the cytosol and subsequently back to the surrounding medium is restricted so that the MitoSOX matrix accumulation is not influenced by $\Delta\Psi_p$. Another explanation may stem from the inability of the existing burst of action potentials to attract a cation from the matrix.

The other verification was based on a survey of MitoSOX Red fluorescence signal changes in the presence of the K_{ATP} opener cromakalim after the addition of glucose to reach 25 mM (Fig. S3E). Despite the fact that cromakalim should keep K_{ATP} always open, hence, despite the absence of plasma membrane depolarization in this case, MitoSOX Red responses were identical with and without cromakalim. These responses illustrated the typical decrease in J_m rate upon high (25 mM) glucose addition, which is the subject of our article. We thus demonstrated a practical

independence on $\Delta\Psi_p$ of MitoSOX Red confocal microscopy monitoring of superoxide release into the mitochondrial matrix.

Figure S3

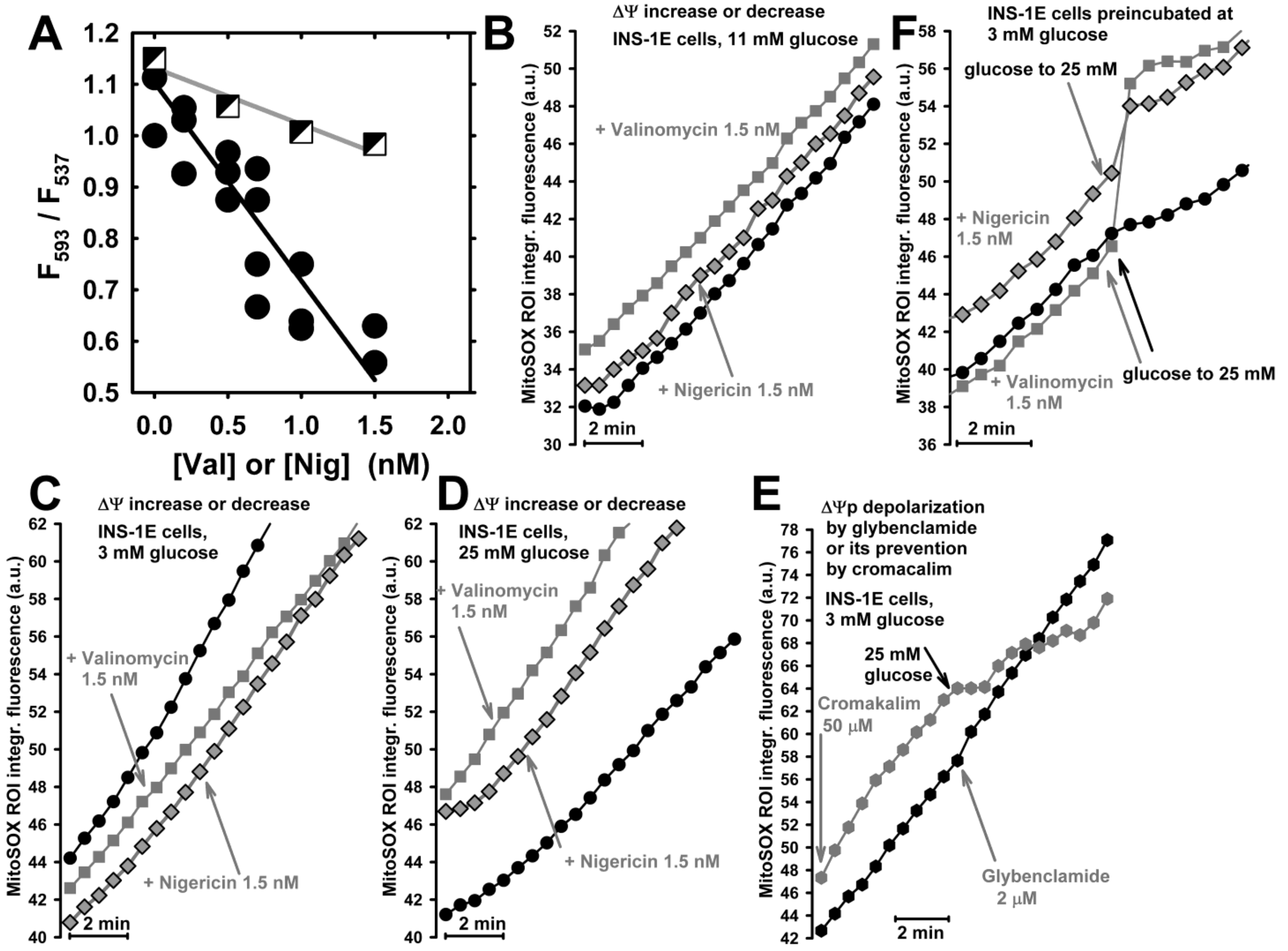

Fig. S3. Independence of MitoSOX Red fluorescence responses on mitochondrial and plasma membrane potential changes during confocal microscopy monitoring

Model pancreatic β-cells, INS-1E cells were assayed for: **A)** $\Delta\Psi_m$ by confocal fluorescence monitoring with the ratiometric probe JC-1. The $\Delta\Psi_m$ decrease experimentally set by varying valinomycin doses (*circles*), is proportional to the decrease in the ratio of JC-1 fluorescence at 593 nm *vs.* 537 nm. A similar titration with nigericin (*squares*) indicated a marginal $\Delta\Psi_m$ decrease.

B–F) Mitochondrial matrix superoxide release monitored by time-lapsed confocal microscopy of MitoSOX Red fluorescence. At each time point, the integral fluorescence from the ROI identical to the mitochondrial network is taken. Traces are illustrated for increasing MitoSOX Red integral fluorescence plotted vs. time for each series of the corresponding confocal images (17, 27, 28) at 11 mM (**A**), 3 mM (**B**, **E** *black*, **F**), and 25 mM glucose (**C**, **E** *gray*) in the absence or presence of 1.5 nM valinomycin (*gray squares*) or 1.5 nM nigericin (*gray diamonds*); or in the presence of 2 μM glibenclamide (**E** *black hexagons*) or 50 μM cromakalim (**E** *gray hexagons*). Glucose or agents were added as indicated by the arrows.

Theoretical calculations of distribution of MitoSOX$^+$ cation between the cellular and mitochondrial compartments

In this section, we justify and interpret the above finding of the approximate MitoSOX fluorescence independence on $\Delta\Psi_m$. For a mitochondrion, the protonmotive force Δp is defined as the electrochemical proton gradient at pH 7 normalized by the Faraday constant, which enables Δp to be expressed in millivolts:

$$\Delta p = \Delta\Psi_m - 61\ \Delta pH \quad \{1\}$$, where

$$\Delta\Psi_m = (2.3RT/F)\ \log_{10}\left\{ [H^+]_{ICS} / [H^+]_m \right\} \quad \{2\}$$

The usual magnitude of ΔpH is about 0.3–0.5, hence at the usual Δp of 200 mV, $\Delta\Psi_m$ is about 170–182 mV. In general, an electric potential component is given by the difference in the concentrations of e.g. the X^+ ion at the two membrane sides 1 and 2 (interfaces) as:

$$\Delta\Psi = (2.3RT/F)\ \log_{10}\left\{ [X^+]_1 / [X^+]_2 \right\} \quad \{3\}$$

For a difference in concentration of one and three orders of magnitude for a permeant cation X^+ at the membrane/water interface, one obtains $\Delta\Psi$ of 61 mV and 183 mV, respectively, at 37 ^{o}C.

Considering plasma membrane potential $\Delta\Psi_p$ in pancreatic β-cells at low (e.g. 3 mM) glucose, the usual value of -70 mV is reported, negative at the cytosolic membrane surface (lipid bilayer leaflet) (17, 27, 28). The hydrophobic and membrane freely permeable cationic MitoSOX$^+$ molecule is therefore accumulated approximately 10-fold in the cell cytosol according to equation {3}. Taking into account further redistribution from the cytosolic (in fact from the intracristal space at the inner mitochondrial membrane), a typical $\Delta\Psi_m$ enables further accumulation of the hydrophobic MitoSOX$^+$ cation in the matrix space by another three orders of magnitude (according to equation {2}). In total, there would be four orders of magnitude higher accumulation in the matrix vs. the extracellular medium. Upon the addition of a high (e.g. up to 25 mM) concentration of glucose, the blockage of K_{ATP} leads to depolarization (17, 27, 28) up to zero mV. Therefore, for a freely penetrating cation, distribution would return to the three orders of magnitude difference, if there was no change in $\Delta\Psi_m$. However, β-cell glucose metabolism and the resulting enhanced proton pumping with the unmatched (slower) re-entry of protons via the rotor of the ATP-synthase leads to the $\Delta\Psi_m$ increase. If one assumes an increase in $\Delta\Psi_m$ by a difference $\delta\Delta\Psi_m$ of 18.4 mV or 10 mV and 5 mV, the cation accumulation in the matrix would further increase 2-fold or 1.45-fold and 1.2-fold, respectively. The distribution of the freely permeant MitoSOX$^+$ cation after the addition of glucose to β-cells thus would only return by a factor of 5 and 6.89 and 8.3 times, respectively, and not 10 times.

However there are two other aspects to be considered. Firstly, due to the lack of super-resolution for the employed confocal microscopy, the signal from mitochondrial ROI (from the mitochondrial network) is a mix of the intracristal space signal and the matrix signal. Assuming an equal mix of these signals, such as occur in cells with rich cristae, the matrix fluorescence signal change at low glucose will in fact be diminished by approximately half. The equation for the MitoSOX$^+$ cation signal from the mitochondrial ROI, given by a n-fold accumulation with regard to the external concentration, can then be written as:

$F_{Low\ glc}$ ~ Accumulation = cytosolic (ICS) ac. + matrix ac. = $0.5 \cdot 10^{\Delta\Psi p/61} + 0.5 \cdot 10^{(\Delta\Psi p+\Delta\Psi m)/61}$ {4}

The second aspect is concerned with the intercalation of MitoSOX into mtDNA. The intercalated fraction cannot return to the ICS and cytosol upon plasma membrane depolarization. When a fraction *D* is defined as the portion of MitoSOX molecules intercalated into mtDNA, equation {4} can be rewritten as follows:

Accumulation = $0.5 \cdot 10^{\Delta\Psi p/61} + 0.5 \cdot (1 - D) \cdot 10^{(\Delta\Psi p+\Delta\Psi m)/61} + 0.5 \cdot D \cdot 10^{(\Delta\Psi p+\Delta\Psi m)/61}$ (fold) {4a}

After the addition of glucose, when the potential increases by a (positively taken) difference $\delta\Delta\Psi_m$ and the plasma membrane is depolarized by the extent of $\delta\Delta\Psi_p$, the "cytosolic" i.e. the intracristal space concentration will be diminished by a factor of $0.5 \cdot 10^{\delta\Delta\Psi p/61}$ and the contribution to the fluorescence signal will be approximately 0.5 arbitrary units, whereas both matrix free and bound fractions will increase by a factor of $0.5 \cdot 10^{\delta\Delta\Psi m/61}$ (in this case multiplied by about 5000). Obviously, the matrix signal predominates in this case. As a result the fluorescence signal given by the accumulation of hydrophobic $MitoSOX^+$ cation in high-glucose-loaded INS-1E cells will be given as:

$F_{High\ glc}$ ~ $0.5 \cdot 10^{\delta\Delta\Psi p/61} + 0.5 \cdot (1 - D) \cdot 10^{(\Delta\Psi p - \delta\Delta\Psi p + \Delta\Psi m + \delta\Delta\Psi m)/61} + 0.5 \cdot D \cdot 10^{(\Delta\Psi p + \Delta\Psi m + \delta\Delta\Psi m)/61}$ (fold) {5}

For a few values of increase in $\delta\Delta\Psi_m$ and various *D* values, one can calculate data, such as those summarized in Table S1A, S1B, illustrating under which conditions the MitoSOX Red fluorescence signal is sensitive to potential changes occurring upon glucose addition and when such sensitivity is quite low.

Table S1 Parts **A**,**B**, illustrate conditions for different values of ***D*** (a fraction of molecules intercalated into mtDNA) under which the MitoSOX Red fluorescence signal is sensitive to potential changes occurring upon glucose addition and when such sensitivity is quite low (*gray background*). Part **A** was calculated for an IMM potential increase of 10 mV, part **B** for 5 mV.

$\Delta\Psi p$	$\delta\Delta\Psi p$	$\Delta\Psi m$	$\delta\Delta\Psi m$	***D***	Signal after glucose	Signal with low glucose
70	60	170	10	**0.05**	**933**	**4306**
70	60	170	10	**0.1**	**1214**	**4306**
70	60	170	10	**0.2**	**1776**	**4306**
70	60	170	10	**0.3**	**2338**	**4306**
70	60	170	10	**0.4**	**2900**	**4306**
70	60	170	10	**0.5**	**3462**	**4306**
70	60	170	10	**0.6**	**4024**	**4306**
70	60	170	10	**0.7**	**4586**	**4306**
70	60	170	10	**0.8**	**5148**	**4306**
70	60	170	10	**0.9**	**5710**	**4306**
70	60	170	10	**1.0**	**6272**	**4306**

$\Delta\psi p$	$\delta_{\Delta\psi p}$	$\Delta\psi m$	$\delta_{\Delta\psi m}$	***D***	Signal after glucose	Signal with low glucose
70	60	170	5	**0.05**	**773**	**4306**
70	60	170	5	**0.1**	**1005**	**4306**
70	60	170	5	**0.2**	**1471**	**4306**
70	60	170	5	**0.3**	**1936**	**4306**
70	60	170	5	**0.4**	**2401**	**4306**
70	60	170	5	**0.5**	**2867**	**4306**
70	60	170	5	**0.6**	**3332**	**4306**
70	60	170	5	**0.7**	**3797**	**4306**
70	60	170	5	**0.8**	**4262**	**4306**
70	60	170	5	**0.9**	**4728**	**4306**
70	60	170	5	**1.0**	**5193**	**4306**

Typical protocol for MitoSOX Red confocal monitoring – is illustrated in the Fig.S3G, when insulinoma cells, preincubated with 3 mM glucose for 1 hr were first equilibrated with MitoSOX Red for 15 min and subsequently confocal monitoring started (time zero, equal to T1 in Fig.S3G). Images were collected each 30 s. At 10 min, glucose was elevated to 25 mM concentration and another time lapsed confocal monitoring continued, again taking an image each 30 s.

Figure S3G. Typical protocol for MitoSOX Red confocal monitoring of mitochondrial superoxide release
Top: Confocal images taken at zero, 10 min and 20 min, while originally 3 mM glucose was set to 25 mM at 10 min. Bottom: resulting trace represents integral fluorescence intensity of MitoSOX Red from mitochondrial ROI of series of confocal images (red), while the two derived slopes are indicated for calculation of J_m rate at 3 mM glucose and J_m rate at 25 mM glucose, respectively.

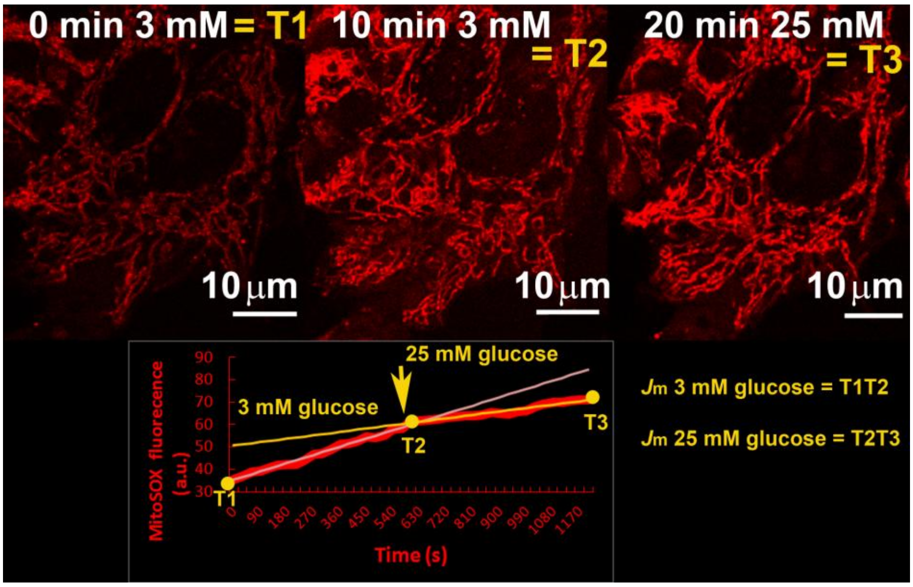

Finally, for isolated pancreatic islets (PI), semiquantification of mitochondrial matrix superoxide release can be done on the basis of MitoSOX Red spectra (Fig. S3H).

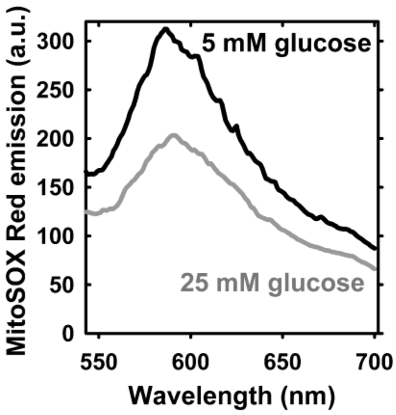

Figure S3H. Changes in MitoSOX Red spectra after glucose addition to isolated mouse PIs

The PIs were isolated from C57Bl6/J mice.

Part IV. Supplemental Results – Constant MnSOD and total SOD activity during the acquisition with MitoSOX Red

Next we estimated possible changes in MnSOD and cell total SOD activities for routinely cultivated INS-1E cells and INS-1E cells preincubated for 15 hrs with 3 mM glucose, both prior to and after addition of glucose to reach 25 mM. Data show that superoxide dismutase activities remained constant during the acquisition time of MitoSOX Red assay.

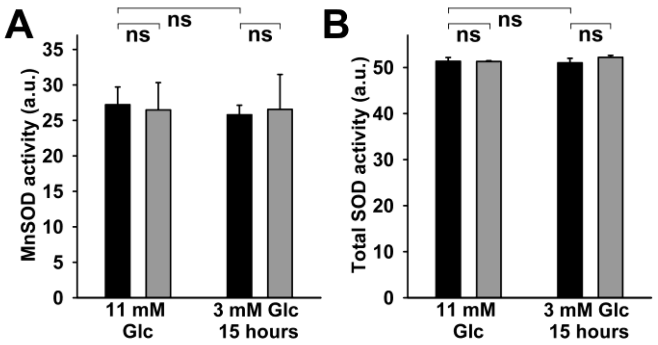

Figure S4. A) MnSOD activity, B) total SOD activity

The MnSOD activity and total SOD activity was assayed (see Methods) before (black columns) and 15 min after glucose addition (*gray columns*). ANOVA (n=4): "ns" non-significant.

Part V. Supplemental Results – Validation of MitoSOX by comparison with MitoB

Further, within the same assay we compared the responses of MitoSOX and LC-MS analyses of the MitoP/(MitoB+MitoP) ratios after 2hr-incubations with the indicated glucose concentrations (0, 3, 11 and 20 mM). MitoSOX confocal fluorescence monitoring was performed after 2 hr for 10 min acquisition and the resulting J_m rates were calculated. Both J_m rates as well as accumulated were normalized to values for 3 mM glucose.

Figure S5. Comparison of MitoSOX responses with LC-MS quantification of accumulated matrix ROS

J_m rates are normalized to those obtained during a 2hr-incubation with 3 mM glucose, after which 10 min of confocal monitoring was performed. MitoP/(MitoB+MitoP) ratios were also quantified also after 2hr-incubations with the indicated glucose concentrations. ANOVA (n = 6): *** p < 0.001; ** p < 0.05.

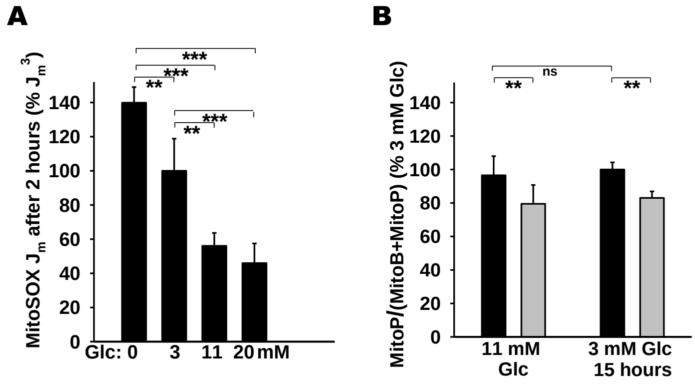

Part VI. Blockage of CitC and 2OGC prevents declines in 2hr-accumulation of ROS in isolated pancreatic islets

The lack of declines in 2hr-ROS accumulation in isolated mouse pancreatic islets is indicated with BTC and *n*-butylmalonate ("Bmln"), inhibiting CitC and 2OGC, respectively.

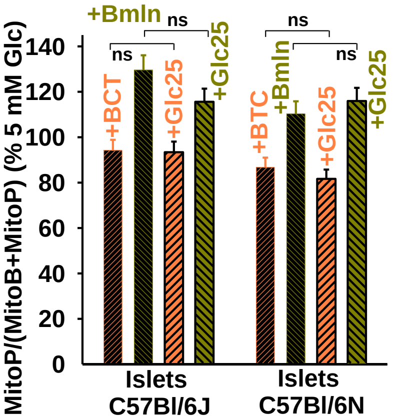

Figure S6. Accumulated ROS in mitochondrial matrix of PI cells during 2 hrs after rising glucose from 5 to 25 mM with inhibited citrate and 2-oxoglutarate carrier

Samples of pancreatic islets isolated from designated mice before (*black dashed*) and after rising glucose to 25 mM („+Glc25") with the indicated inhibitors. Ratios were normalized to values obtained for incubations with 5 mM glucose without any agents. ANOVA (n = 4) „ns", non-significant. 10 mM BTC and 5 mM *n*-butylmalonate were used.

Part VII. Increase in cytosolic NADPH$_F$ as monitored using the iNAP1 fluorescent probe – blockage of CitC and 2OGC

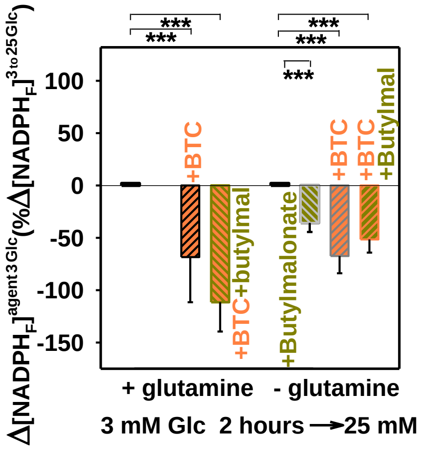

Figure S7. Cytosolic NADPH elevations assayed by the iNAP1 fluorescence probe in INS-1E cells (preincubated with 3 mM glucose for 2 hrs) upon glucose addition to final concentration of 25 mM in the absence or presence of CitC inhibitor (10 mM BTC, orange) or 2OGC inhibitor (5 mM n-butylmalonate, yellow-green) or both. Inhibitors were added prior to the glucose addition. Error bars represent SDs. ANOVA (n = 40–60): *** $p < 0.001$.

Part VIII. 2chFLIM assay for bound NADPH and NADH within mitochondrial ROI

Next we demonstrated that the ratio of bound species $NADPH_B/NADH_B$, derived from 2chFLIM data, did not significantly increase after the elevation of glucose to 25 mM in the matrix (Fig.S8).

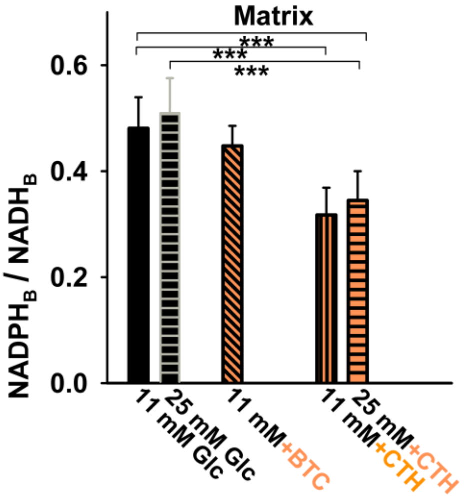

Figure S8. Mitochondrial matrix ratios of bound $NADPH_B/NADH_B$ derived from 2chFLIM. Data were calculated using the integral parameters from the mitochondrial network ROI and expressed as averages ±S.D. of 15 analyzed 2chFLIM NAD(P)H autofluorescence images, each containing 80–100 cells.
